# EHRtemporalVariability: delineating temporal data-set shifts in electronic health records

**DOI:** 10.1093/gigascience/giaa079

**Published:** 2020-07-30

**Authors:** Carlos Sáez, Alba Gutiérrez-Sacristán, Isaac Kohane, Juan M García-Gómez, Paul Avillach

**Affiliations:** Biomedical Data Science Lab, Instituto Universitario de Tecnologías de la Información y Comunicaciones, Universitat Politècnica de València, Camino de Vera s/n, Valencia 46022, España; Department of Biomedical Informatics, Harvard Medical School, Boston, Massachusetts, USA; Department of Biomedical Informatics, Harvard Medical School, Boston, Massachusetts, USA; Department of Biomedical Informatics, Harvard Medical School, Boston, Massachusetts, USA; Biomedical Data Science Lab, Instituto Universitario de Tecnologías de la Información y Comunicaciones, Universitat Politècnica de València, Camino de Vera s/n, Valencia 46022, España; Department of Biomedical Informatics, Harvard Medical School, Boston, Massachusetts, USA; Computational Health Informatics Program, Boston Children’s Hospital, Boston, Massachusetts, USA

**Keywords:** data-set shifts, data quality, temporal variability, scientific data sets, electronic health records, claims data, research repositories, information geometry, visual analytics, R package

## Abstract

**Background:**

Temporal variability in health-care processes or protocols is intrinsic to medicine. Such variability can potentially introduce dataset shifts, a data quality issue when reusing electronic health records (EHRs) for secondary purposes. Temporal data-set shifts can present as trends, as well as abrupt or seasonal changes in the statistical distributions of data over time. The latter are particularly complicated to address in multimodal and highly coded data. These changes, if not delineated, can harm population and data-driven research, such as machine learning. Given that biomedical research repositories are increasingly being populated with large sets of historical data from EHRs, there is a need for specific software methods to help delineate temporal data-set shifts to ensure reliable data reuse.

**Results:**

EHRtemporalVariability is an open-source R package and Shiny app designed to explore and identify temporal data-set shifts. EHRtemporalVariability estimates the statistical distributions of coded and numerical data over time; projects their temporal evolution through non-parametric information geometric temporal plots; and enables the exploration of changes in variables through data temporal heat maps. We demonstrate the capability of EHRtemporalVariability to delineate data-set shifts in three impact case studies, one of which is available for reproducibility.

**Conclusions:**

EHRtemporalVariability enables the exploration and identification of data-set shifts, contributing to the broad examination and repurposing of large, longitudinal data sets. Our goal is to help ensure reliable data reuse for a wide range of biomedical data users. EHRtemporalVariability is designed for technical users who are programmatically utilizing the R package, as well as users who are not familiar with programming via the Shiny user interface.

Availability: https://github.com/hms-dbmi/EHRtemporalVariability/

Reproducible vignette: https://cran.r-project.org/web/packages/EHRtemporalVariability/vignettes/EHRtemporalVariability.html

Online demo: http://ehrtemporalvariability.upv.es/

## Introduction

The widespread adoption of data-sharing technologies, health information standards, and open-data initiatives are inspiring the creation of research data repositories that contain large-scale historical data from electronic health records (EHRs) [1]. These repositories represent a new class of longitudinal, real-world data, defined as large data sets collected over time from sources outside of clinical trials or specific research cohorts. While reuse of this data, ranging from clinical observations to molecular information, has begun to enhance the efficacy and generalization of biomedical and clinical research, efforts towards the efficient and reliable reuse of real-world data are still in early stages [2, 3].

Most recently, researchers from the machine-learning community have identified EHR data as an important source of labeled data with which diagnostic and prognostic models can by constructed [4]. Among the major hurdles in reusing such EHR data, however, is its temporal variability. Indeed, clinical care processes and their local variations are permeated with a variety of batch effects and biases [5–9]. This situation is similar to that in genomics and other “omics” research, where batch effects can be introduced by technical sources of variation that have been added to samples during acquisition handling [10, 11].

Temporal variability artifacts—in the form of data-set shifts—can impact data quality and challenge the secondary use of data, particularly for population and data-driven research [8, 12–14], as well as for machine learning [15, 16]. In addition, the EHRs themselves can contribute to variability, as they reflect the evolution of administrative practice and reimbursement policies, all of which can gradually or abruptly shift over time. For example, updates in coding systems, such as the International Classification of Diseases (ICD) [17], or modifications to clinical guidelines often lead to variable data representations across multiple diseases over time.

To circumvent these issues, researchers have traditionally deployed statistical process control–based methods, which expose the time-points at which reference changes occur. Shewhart and Levey-Jennings charts, for example, have been employed in laboratory quality control efforts [18, 19]. Similarly, autocorrelation or time series–based approaches have been used to uncover periodicity and changes within summary statistics derived from longitudinal samples, such as batched averages [20–23]. When the dates of such reference changes are exposed, statistical tests can uncover significant differences between time periods. However, these approaches tend to promulgate the loss of information, especially when deployed when using highly coded data: for example, in categorical variables with a particularly high number of values, such as when using the ICD Ninth Revision, Clinical Modification (ICD-9-CM), which has over 16,000 distinct codes, as well as in multimodal statistical distributions, in which multiple sub-phenotypes are present.

In the R programming language, there are distinct packages that can help in managing or describing EHRs. For example, the *rEHR* package focuses on querying and filtering, while the *EHR* and *comoRbidity* packages allow the performance of descriptive, Phenome-Wide Association Studies (PheWAS), and comorbidity analyses [24–26]. Other packages, such as *MTS* or *qcc*, allow the performance of time-series or statistical process control–based analyses, which assist in detecting data-set shifts in EHRs [27, 28].

To our knowledge, EHRtemporalVariability is the first package that provides specific data-set shift delineation, which can be used on raw EHRs and other data sources. The key advantage is its suitability for multi-modal and highly coded information, which are common features of biomedical data.

## Materials and Methods

EHRtemporalVariability is designed to explore and identify the temporal variability of categorical and numerical data over time. The app provides the means to visually and analytically delineate data-set shifts in multi-modal and highly coded information. A key advantage is that no distributional assumptions are made. This enables straightforward use, as well as visual analytics on large EHR-coded and numerical variables with no loss of information. In addition, the tool's methodological and iterative use can identify and define reference changes that might otherwise impede further research. Analyses can proceed using both the R package (RRID:SCR_001905) and Shiny app (RRID:SCR_001626) with minimum effort. Data can flow through the pipeline from their initial raw, individual-level state to the final results.

EHRtemporalVariability is based on the probabilistic temporal variability methods that we developed and validated previously [6, 9, 13]; namely, information geometric temporal (IGT) plots and data temporal heat maps (DTHs). We offer these for the first time as an open-source R package and Shiny app. Our method is based upon the estimation and comparison of data statistical distributions over time (see Supplementary Methods online). IGT plots project time batches as a series of points. The distances between them correspond to the dissimilarity of their statistical distributions. This yields an empirical layout of temporal relationships between batches; namely, a non-parametric temporal statistical manifold.

IGT plots allow users to visually identify four types of changes: (i) trends, represented as continuously flowing time batches; (ii) abrupt changes, shown as gaps between groups of batches; (iii) temporal subgroups, depicted as clusters of batches; and (iv) seasonality, portrayed as temporal cycles. Batches are labeled by date and color-coded to distinguish seasonal effects. Additionally, IGT plots can include a smoothed trajectory of the information evolution over time. The IGT plot data also provides the means to identify those changes in order to model seasonal effects or apply clustering methods to depict temporal subgroups [9]. Complementing the IGT plots, DTHs allow users to explore changes in absolute and relative frequencies over time and, simultaneously, at multiple variable values (e.g., frequencies of phenotypes).

**Figure 1:**
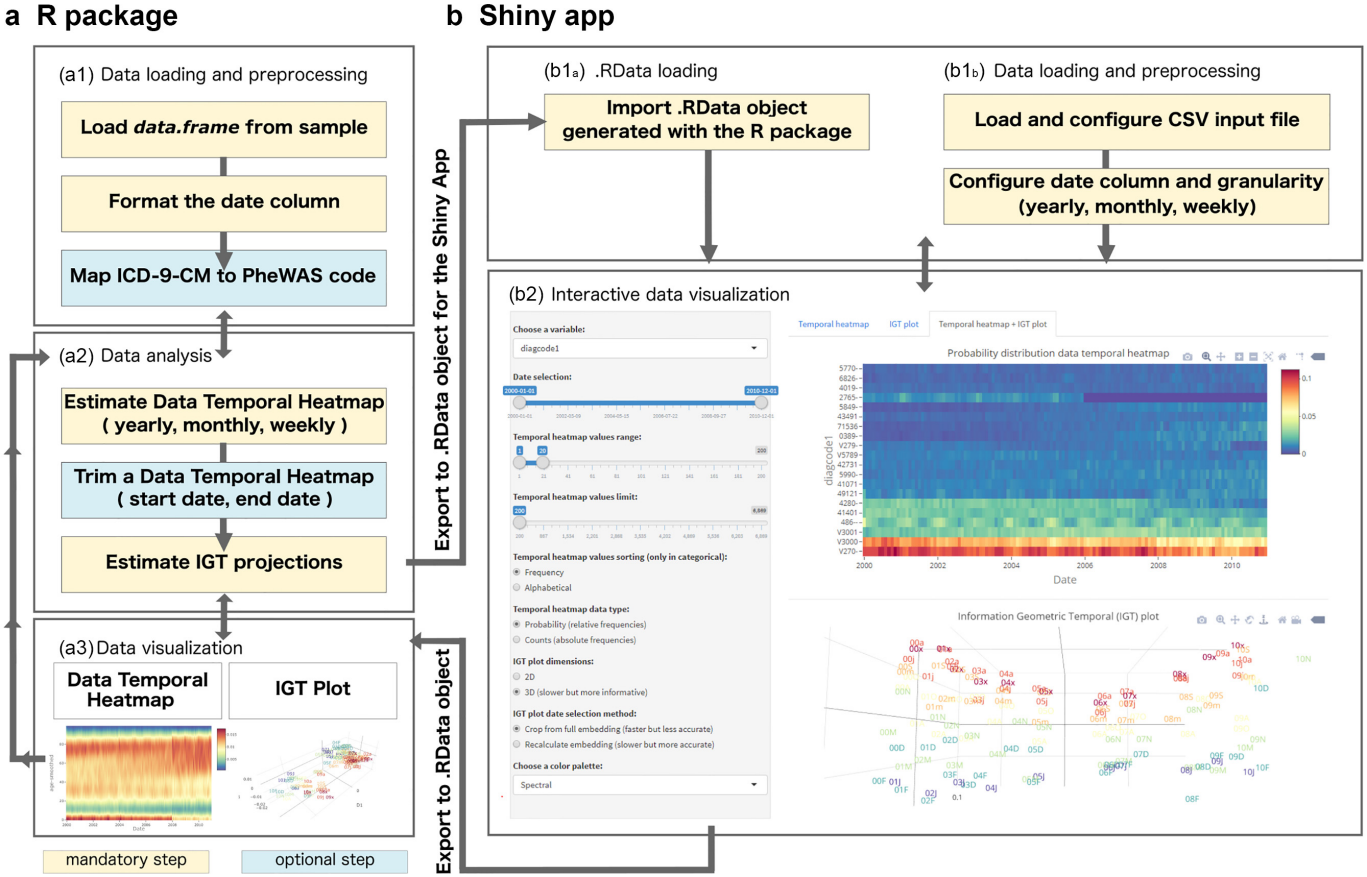
EHRtemporalVariability R package (**a**) and Shiny app (**b**) outline. The general workflow of the R package is organized as a set of functions for: (a1) data loading and preprocessing, (a2) data analysis, and (a3) data visualization. The main input is an R *data.frame*, in which one column defines the reference date. The classes of the remaining columns determine the variable's treatment for distribution estimation and plotting during analysis and visualization. (See Supplementary Methods section online.) Specifically, “factor” and “character” receive categorical treatment, while “numeric,” “integer,” and “date” receive numerical treatment. The DTH object estimation takes the input “data.frame” and analysis parameters. These include temporal granularity; predefined distribution support (a range of possible values or bins for each variable, auto-calculated from data by default); handling of missing batches; and the choice of whether to smooth distributions in numerical variables. The DTH can be trimmed by values and date range. The IGT projection estimation takes as input the DTH and the desired number of dimensions for embedding. The DTH can be plotted as a dynamic Plotly (RRID:SCR_013991) heat map, in which the color of each cell indicates the frequency (relative or absolute) at a specific date batch (column) for the value of a variable (categorical and numerical integer) or range or values (numerical continuous). IGT plots can be visualized as either 2- or 3-dimensional dynamic Plotly plots. The input for the Shiny app can be either an .RData object exported from the R package (b1_A_) or a raw .csv input file (b1_B_). The Shiny app provides an interactive dashboard (b2) for controlling the visualization parameters of the programmatic R functions. This is done via reactive sliders, selection boxes, and buttons. These have a direct effect on heat maps and IGT plots. Further, we include different color palettes suited for different types of color-blindness. For further information about all the EHRtemporalVariability functionality, see https://cran.r-project.org/web/packages/EHRtemporalVariability/vignettes/EHRtemporalVariability.html.

Overall, the EHRtemporalVariability R package (Fig. 1a) and Shiny app (Fig. 1b) provide a set of functionalities that allow users to perform three actions: loading and processing data sets; running batched data analyses for the estimation of DTHs and IGT projections; and visualizing these data through interactive plots. The R package also enables users to conduct these tasks programmatically, enabling more flexibility in data processing and further analysis of the resultant objects and embedding matrices.

The Shiny app provides a graphical user interface with two objectives. First, users unfamiliar with R programming can load .csv files and easily produce and visualize their results, which can be exported as an .Rdata file for further inspection in R. Second, we provide an exploratory, dynamic dashboard to improve the user experience, enabling a means to load results exported from the R package as an .RData file. We customized both the R package and Shiny app visualizations for users who are color-blind.

A more detailed description of methods is available in the Supplementary Material online.

## Results

We validated the functionality of EHRtemporalVariability using 3 case studies. The first involved the i2b2 (Informatics for Integrating Biology & the Bedside) Boston Children's Hospital Autism Spectrum Disorders cohort (BCH-ASD), including 12,000 patients (1.2 M ICD-9-CM clinical observations) whose data were recorded from 1981 to 2016. This project was reviewed by the Boston Children's Institutional Review Board.

**Figure 2:**
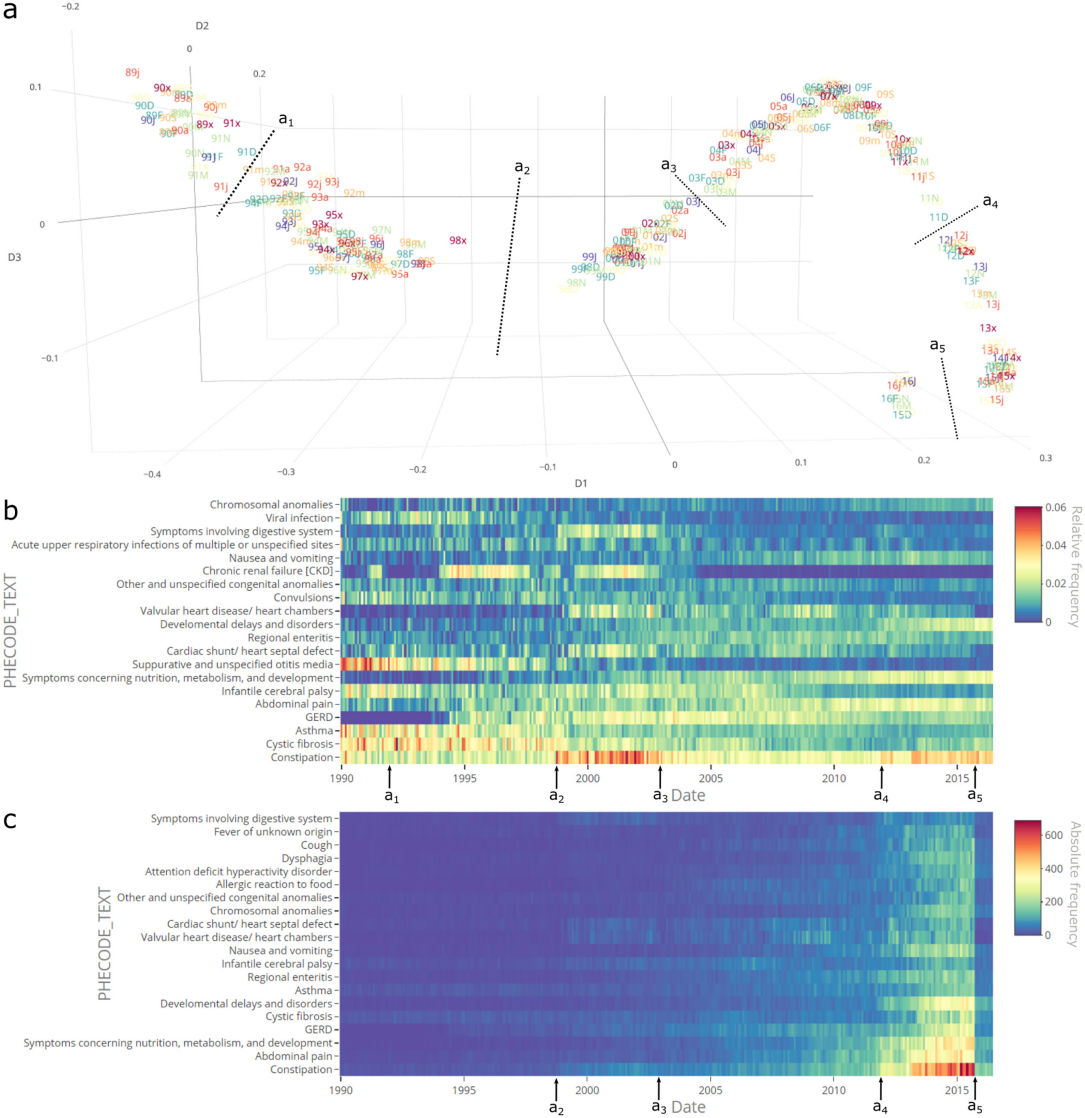
Delineation of data-set shifts in the BCH-ASD's EHR historical clinical observations. (**a**) IGT plot describing the evolution of distributions of ICD-9-CM codes over time: specifically, monthly time batches taken from March 1989 to June 2016. The projection of time batches is based on embedding the dissimilarities among their distributions using multidimensional scaling. The IGT plot axes corresponds to the three first temporal components of variance. Several slight abrupt changes are apparent during October 1991 (a_1_), January 2003 (a_3_), and December 2011 (a_4_). Major abrupt changes appear during October 1998 (a_2_) and October 2015 (a_5_). Overall, there is a trend in the distribution changes across the entire time frame. Text labels are formatted as yym, where yy is a 2-digit year and m is an abbreviated month, with the months displayed as {“J,” “F,” “M,” “A,” “m,” “j,” “x,” “a,” “S,” “O,” “N,” “D”}. (**b**) DTH of the 20 most frequent relative frequencies of PheWAS codes text. (**c**) DTH of the 20 most frequent absolute frequencies of PheWAS codes text. The major driver for (a_2_) was a decrease in “other symptoms” and “other tests” codes. Thus, we excluded these to investigate the effects on comorbidities and obtained (b) and (c). Changes in October 1998 (a_2_) include increases in the frequencies of constipation, major depressive disorder, symptoms involving digestive system, and type 2 diabetes. Other minor decreases included cystic fibrosis, other diseases of blood and blood forming-organs, and type 1 diabetes, among “others." As observed in (c), some of the delineated changes are time-correlated with alterations in absolute frequencies.

In this cohort, the IGT plot uncovered five abrupt changes of reference (Fig. 2a). The most obvious was in billing codes, for which frequencies changed in October 1998 (Fig. 2a-a_2_). Accordingly, we discovered an abrupt change in the relative frequencies of ICD-9-CM codes during that month. Specifically, the DTH of the ICD-9-CM codes (Supplementary Material Fig. 1) showed an abrupt decrease in the frequency of codes 780 (general symptoms), 780.9 (other general symptoms), and 289.9 (unspecified diseases of blood and blood-forming organs). We also tracked increases in more specific 780.x codes, 296.x codes (episodic mood disorders), and other trends, which are represented as gradual changes.

While investigating the root cause of the October 1998 reference change, we found that it coincided with a yearly ICD-9-CM update. However, there was no apparent relationship between the documented changes and our findings.

To further investigate this variability, we mapped ICD-9-CM codes to PheWAS codes [29]. We removed all the observations listed as “other symptoms” and “other tests.” Still, the abrupt change persisted even when we delineated changes for further specific comorbidities (Fig. 2b and c). Intriguingly, the absolute number of observations also increased at the start of the month. Although this reference change appears to have been motivated by a systemic or protocol change, the exact cause remains unclear. We suggest that this reference change is a potential data-set shift that should be considered in any future BCH-ASD data analysis.

**Figure 3:**
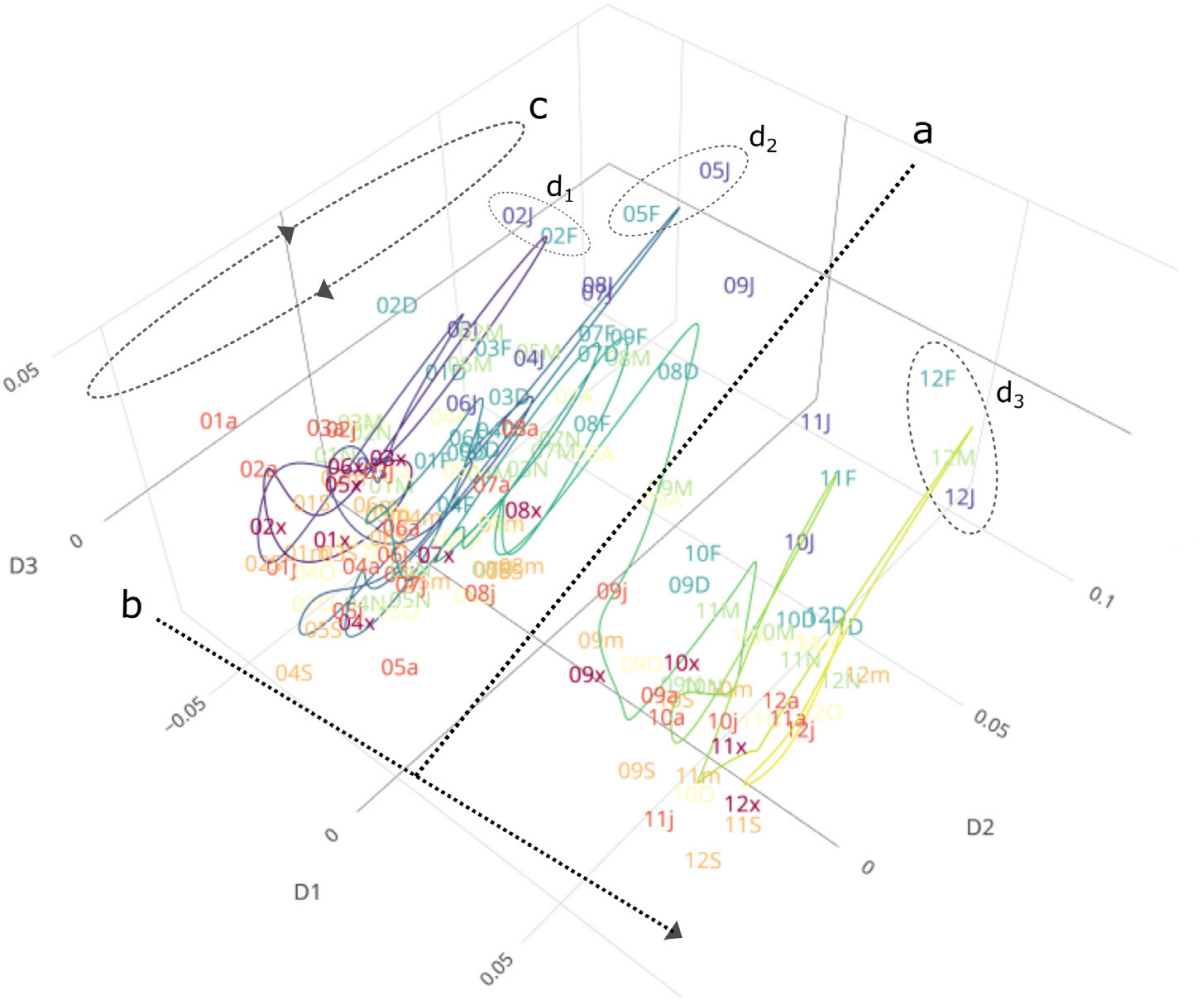
IGT plot of the basic cause of death in the mortality registry of the region of Valencia, Spain, coded with ICD Tenth Revision Mortality Causes List 1. (**a**) The major abrupt change associated with the update of the national certificate of death is depicted as a dotted line that splits the main trend through the entire period of study (**b**), a trend that lays in dimension D1. (**c**) Yearly seasonality of causes of death, highlighted by coloring scheme and trajectory cycles and laid out across dimension D2. (**d_1_, d_2_, d_3_**) Flu epidemics in 2002, 2005, and 2009 as outlying batches and showing fast trajectory deviations. Text labels are formatted as yym, where yy is a 2-digit year and m is an abbreviated month, with the months displayed as {'J,” “F,” “M,” “A,” “m,” “j,” “x,” “a,” “S,” “O,” “N,” “D”}. The drivers for (a) included a relatively abrupt decrease in “symptoms, signs, and abnormal clinical and laboratory findings, not elsewhere classified” and an increase in “hypertensive diseases,” among others.

The second case study replicates a baseline experiment we previously performed using the mortality registry of Valencia, Spain [13]. The registry recorded 512,000 deaths between 2000 and 2012. Similar to the Boston Children's results, the registry's statistical distributions changed abruptly in 2009, following an update in the fields of the Spanish national death certificate. Notably, this reference change impacted the “basic cause of death," a variable used for reporting national and international death statistics (Fig. 3a and Supplementary Material Fig. 2). This occurred even after the variable was retrospectively corrected. The results also showed an overall trend through the entire period of the study (Fig. 3b), a yearly seasonality of causes of death (Fig. 3c), and spotted outlier months associated with flu epidemics in 2002, 2005, and 2009 (Fig. 3d_1_, d_2_ and d_3_, respectively).

Finally, we validated EHRtemporalVariability with the National Hospital Discharge Survey (NHDS)—an open data set that includes 3.25 million inpatient discharges from US hospitals (2000–2010)—and both demographic and ICD-9-CM–coded data. Again, we uncovered several abrupt changes throughout multiple variables [6, 9], including the recoding of discharge age in 2008; ICD-9-CM diagnoses (Fig. 1); procedure codes; and yearly abrupt changes in diagnosis-related group codes. These findings were in addition to the expected context-induced trends and seasonality. After mapping the NHDS ICD-9-CM codes to PheWAS codes, notable changes remained, including in October 2007, coincident with the yearly ICD-9-CM update. Note that this case study is available for replication within the package and Shiny app demonstration at http://ehrtemporalvariability.upv.es/ and in the*GigaScience* Database [30], and a tutorial on how to interpret temporal changes in IGT plots using NHDS data is available in the package vignette. Performance measures for the three case studies are described in the Supplementary Material.

## Discussion

In light of the changes uncovered by EHRtemporalVariability, we argue that users of the package can more accurately repurpose their data analyses. For example, in the presence of abrupt changes, one can compare the performance of predictive modeling using only the most recent temporal subgroups versus full data inclusion.

In addition, incremental learning approaches can also be adopted to deal with abrupt changes and continuous trends in machine learning, as can introducing seasonal or subgroup-related effects on models. Finally, in cases of descriptive analyses, such as those in PheWAS studies, we suggest evaluating the possible effects of temporal changes in results by making separate analyses at distinct temporal subgroups, as opposed to performing a more global analysis.

## Conclusions

In conclusion, EHRtemporalVariability is a data quality assessment tool that enables the broad exploration and repurposing of large data sets collected over time. We view the app as a key stepping stone toward the identification of data-set shifts for data reuse, specifically in machine learning. Target users are biomedical data scientists and bioinformaticians, as well as epidemiologists and hospital data managers. The tool can assist in exploring the effects of system, protocol, and environment-induced changes on data. We also encourage the use of EHRtemporalVariability to analyze the impact of the adoption of new coding systems, such as the ICD Tenth Revision [31]. EHRtemporalVariability can be used on any additional coded and numerical data modalities and, because it is open source, the app can be extended with new functionality or uses by the scientific community.

## Availability of source code and requirements

- Project name: EHRtemporalVariability
- Project home page: https://github.com/hms-dbmi/EHRtemporalVariability/
- Operating system(s): Platform independent
- Programming language: R
- Other requirements: R 3.3.0, dplyr, plotly, shiny, zoo, xts, lubridate, RColorBrewer, viridis, scales, methods, MASS
- License: Apache License 2.0
- CRAN repository: https://cran.r-project.org/package=EHRtemporalVariability
- bio.tools ID: biotools: ehrtemporalvariability
- SciCrunch ID: RRID:SCR_018663
- Shiny app repository: https://github.com/hms-dbmi/EHRtemporalVariability-shiny
- Reproducible vignette: https://cran.r-project.org/web/packages/EHRtemporalVariability/vignettes/EHRtemporalVariability.html
- On-line Shiny app demo (for privacy reasons loading raw .csv data is disabled): http://ehrtemporalvariability.upv.es/

## Availability of supporting data and materials

The data of the National Hospital Discharge Survey case study are publicly available at https://www.cdc.gov/nchs/nhds/index.htm. A random subset of this data-set is available as a proxy for testing purposes within the EHRtemporalVariability package, and reproducible examples are available within the package help, its vignette, and the online demo (http://ehrtemporalvariability.upv.es/). An archival snapshot of the code is available in the*GigaScience* GigaDB repository [30]. Access to Boston Children's Hospital Autism Spectrum Disorders cohort case study data is restricted by Boston's Children's Institutional Review Board. Access to the Mortality case study data is restricted by the Conselleria de Sanitat Universal i Salut Pública, Generalitat Valenciana, Spain.

## Abbreviations

BCH-ASD: Boston Children's Hospital Autism Spectrum Disorders cohort; DTH: data temporal heat map; EHR: electronic health record; ICD: International Classification of Diseases; ICD-9-CM: ICD Ninth Revision, Clinical Modification; IGT: Information Geometric Temporal; NHDS: National Hospital Discharge Survey; PheWAS: Phenome-Wide Association Studies

## Competing interests

The authors declare that they have no competing interests.

## Funding

This work was supported by Universitat Politècnica de València grant PAID-00–17, Generalitat Valenciana grant BEST/2018, and projects H2020-SC1–2016-CNECT No. 727560 and H2020-SC1-BHC-2018–2020 No. 825750.

## Author contributions

C.S., A.G.S., J.M.G.G. and P.A. conceived the R package. C.S., A.G.S., I.K. and P.A. conceived the BCH case study. C.S. and J.M.G.G. conceived the original methods and NHDS and Mortality case studies. C.S. programmed the R temporal variability analysis methods and plots. C.S. and A.G.S. programmed the R package wrapper, data loading and pre-processing functions, Shiny app, and wrote the documentation. C.S. and A.G.S. performed data collection, processing and analysis of the BCH case study. C.S. and J.M.G.G. performed data processing and analysis of the NHDS and Mortality case studies. C.S., A.G.S., I.K., J.M.G.G. and P.A. reviewed and interpreted the results. C.S. drafted the article. C.S. and A.G.S. drafted the figures. C.S., A.G.S., I.K., J.M.G.G. and P.A. provided critical revision of the article and approved the final version to be publishedC.S., A.G.S., J.M.G.G. and P.A. conceived the R package. C.S., A.G.S., I.K. and P.A. conceived the BCH case study. C.S. and J.M.G.G. conceived the original methods and NHDS and Mortality case studies. C.S. programmed the R temporal variability analysis methods and plots. C.S. and A.G.S. programmed the R package wrapper, data loading and pre-processing functions, Shiny app, and wrote the documentation. C.S. and A.G.S. performed data collection, processing and analysis of the BCH case study. C.S. and J.M.G.G. performed data processing and analysis of the NHDS and Mortality case studies. C.S., A.G.S., I.K., J.M.G.G. and P.A. reviewed and interpreted the results. C.S. drafted the article. C.S. and A.G.S. drafted the figures. C.S., A.G.S., I.K., J.M.G.G. and P.A. provided critical revision of the article and approved the final version to be published.

## Supplementary Material

giaa079_GIGA-D-19-00376_Original_Submission

giaa079_GIGA-D-19-00376_Revision_1

giaa079_Response_to_Reviewer_Comments_Original_Submission

giaa079_Reviewer_1_Report_Original_Submission Jing Zhao -- 1/24/2020 Reviewed

giaa079_Reviewer_2_Report_Original_Submission Emre Guney -- 4/26/2020 Reviewed

giaa079_Reviewer_2_Report_Revision_1 Emre Guney -- 6/12/2020 Reviewed

giaa079_Supplemental_Files

## Supplementary Data

Supplementary data include: (1) the technical details of the methods, (2) supplementary figures, and (3) a performance measures test.
