# Supplementary material for: EHRtemporalVariability: delineating temporal data-set shifts in electronic health records
Source: Gigascience. 2020 Jul 30;9(8):giaa079. doi: 10.1093/gigascience/giaa079 (PMC7391413; doi:10.1093/gigascience/giaa079)
Supplement: giaa079_GIGA-D-19-00376_Revision_1 [file giaa079_giga-d-19-00376_revision_1.pdf]

GigaScience

EHRtemporalVariability: delineating temporal dataset shifts in electronic health records

--Manuscript Draft--

**Manuscript Number:**	GIGA-D-19-00376R1
**Full Title:**	EHRtemporalVariability: delineating temporal dataset shifts in electronic health records
**Article Type:**	Technical Note
**Funding Information:**	Universitat Politècnica de València (PAID-00-17) — Dr Carlos Sáez<br>Conselleria d'Educació, Investigació, Cultura i Esport (BEST/2018) — Dr Carlos Sáez<br>H2020 Societal Challenges (H2020-SC1-2016-CNECT No. 727560) — Dr Juan M García-Gómez<br>H2020 Societal Challenges (H2020-SC1-BHC-2018-2020 No. 825750) — Dr Juan M García-Gómez
**Abstract:**	Background<br>Temporal variability in healthcare processes or protocols is intrinsic to medicine. Such variability can potentially introduce dataset shifts, a data quality issue when reusing electronic health records (EHRs) for secondary purposes. Temporal dataset shifts can present as trends, as well as abrupt or seasonal changes in the statistical distributions of data over time. The latter are particularly complicated to address in multi-modal and highly coded data. These changes, if not delineated, can harm population and data-driven research, such as machine learning. Given that biomedical research repositories are increasingly being populated with large historical data from EHRs, there is a need for specific software methods to help delineate temporal dataset shifts to ensure reliable data reuse.<br>Findings<br>EHRtemporalVariability is an Open Source R-package and Shiny-app designed to explore and identify temporal dataset shifts. EHRtemporalVariability estimates the statistical distributions of coded and numerical data over time; projects their temporal-evolution through non-parametric Information Geometric Temporal plots; and enables the exploration of changes in variables through Data Temporal Heatmaps. We demonstrate the capability of EHRtemporalVariability to delineate dataset shifts in three impact case studies, one of them available for reproducibility.<br>Conclusions<br>EHRtemporalVariability enables the exploration and identification of dataset shifts, contributing to the broad examination and repurposing of large, longitudinal datasets. Our goal is to help ensure reliable data reuse for a wide range of biomedical data users. EHRtemporalVariability is designed for technical users who are programmatically utilizing the R-package, as well as users who are not familiar with programming via the Shiny user interface.<br>Availability: https://github.com/hms-dbmi/EHRtemporalVariability/<br>Reproducible vignette:<br>https://cran.rproject.org/web/packages/EHRtemporalVariability/vignettes/EHRtemporalVariability.html<br>On-line demo: http://ehrtemporalvariability.upv.es/
**Corresponding Author:**	Carlos Sáez, Ph.D.<br>Universidad Politecnica de Valencia<br>València, València SPAIN
**Corresponding Author Secondary Information:**	
**Corresponding Author's Institution:**	Universidad Politecnica de Valencia
**Corresponding Author's Secondary Institution:**	
**First Author:**	Carlos Sáez, Ph.D.

Powered by Editorial Manager® and ProduXion Manager® from Aries Systems Corporation

First Author Secondary Information:	
Order of Authors:	Carlos Sáez, Ph.D.
	Alba Gutiérrez-Sacristán, Ph.D.
	Isaac Kohane, M.D., Ph.D.
	Juan M García-Gómez, Ph.D.
	Paul Avillach, M.D., Ph.D.
Order of Authors Secondary Information:	

Response to Reviewers:

Dear Editor,

Please find enclosed our revised version of the manuscript entitled, "EHRtemporalVariability: delineating temporal dataset shifts in electronic health records" (GIGA-D-19-00376).

We would like to thank the Editor for the opportunity to resubmit the manuscript to be considered for publication in GIGASCIENCE and also the Reviewers for their comments. We have used them to improve both the manuscript and the application.

Please find below our response to the Reviewers' comments.

Editor

EQ1. In addition, please register any new software application in the bio.tools and SciCrunch.org databases to receive RRID (Research Resource Identification Initiative ID) and biotoolsID identifiers, and include these in your manuscript. This will facilitate tracking, reproducibility and re-use of your tool.

According to the Editor's request, we have registered our software in bio.tools (https://bio.tools/ehrtemporalvariability) and it is approved as a new resource in SciCrunch.org (RRID:SCR_018663). We thank the Editor for making this suggestion; we are sure this will facilitate disseminating this work to end users.

Reviewer reports

Reviewer #1

"The main objective of this technical note is to introduce an R package and a Shiny app that was developed to explore and identify the temporal variability of electronic health record (EHR). To achieve this goal, the authors first gave a brief introduction to the methodologies embedded in the R tools in a way that easy to understand and elucidated the functionalities of these tools. Three case studies were listed afterward. This topic is in-time for promoting the usage of EHR in clinical research. However, it could be further improved if the following comments are soundly fixed.

-We thank the Reviewer for highlighting the timeliness of our work for clinical research. We also expect that the work will help facilitate the performance of reliable Real World Data research using large datasets acquired over long time periods."

R1Q1) "The paper only shows the IGT plot and temporal heatmap, it would be improved if the authors provide more comprehensive analysis functions and visualizations shown in the original case studies, e.g. the kinematic figures, temporal subgroup discovery and clustering validation, the variability of JSD distributions."

In this work—and upon the current release of the tool—we aimed to provide full support on IGT plots and temporal heatmap estimation and visualization. We also

Powered by Editorial Manager® and ProduXion Manager® from Aries Systems Corporation

planned for further versions of the tool, including improved functionality for the kinematic figures described in our IJMI paper and additional subgroup discovery support. However, following the Reviewer's suggestion, we have further added the ability to estimate and plot the kinematic trajectory to the IGT projection. The former is done using the new function “estimateIGTTrajectory” and the latter using the new parameter “trajectory” in the plotIGTProjection function. We refer to the Reviewer to the 2-D sample in the new package Vignette: http://personales.upv.es/carsaesi/EHRtemporalVariability/EHRtemporalVariability.html#trajectory, as well as to the online demo at http://ehrtemporalvariability.upv.es.

In addition, we added an extra example code to the Vignette to validate the temporal clusters using the DBSCAN clustering algorithm. Specifically, we added a new section in the Vignette (Section 5) to help interpreting temporal changes in IGT projections: http://personales.upv.es/carsaesi/EHRtemporalVariability/EHRtemporalVariability.html#example

R1Q2) "The manuscript needs careful editing for English and style. For example, the arrows and result image A1-A3 are not vertically aligned well in Figure 1; the arrow in A2 uses a lighter grey color while the meaning seems no different from others; the different border width of B2; the local version of Shiny app accepts input of three separators as comma, semicolon, and tab, however, the authors only claim the input as comma-separated values file (CSV)."

Responding to the Reviewer’s comment, we have carefully revised Figure 1. We are aware of the CSV claim, however, we chose to use it instead of the alternate delimiter-separated values (DSV), given that CSV is more widely accepted and equivalent in the sense that it also accepts using distinct delimiter values (i.e., semicolons and commas are distinctly used across countries). Finally, the manuscript was revised by a professional proofreading service in American English. To address the Reviewer’s comment specifically, however, we edited an additional time for this revised version.

R1Q3) The abbreviation of National Hospital Discharge Survey in the title of table SM3&6 is incorrect.

We thank the Reviewer for pointing this out. We have fixed the error accordingly.

Reviewer #2:

"The manuscript presents an EHRtemporalVariability, an R package and accompanying Shiny app, that provides several visualization functions of EHR data towards identifying changes in the distribution of features in the data. The package accepts the data as an R data frame and allows generating Information-Geometric-Temporal (IGT) plots and Data Temporal Heatmaps (DTHs) -two types of plots previously developed by the authors— that help visually detecting different type of changes in the data described as trend, abrupt change, temporal subgroup, seasonality in the manuscript. Overall, the article is well written and the package and the app are fairly explained. I list several points that need to be addressed in my opinion."

We thank the Reviewer for comments about our paper and the app.

R2Q1) "The introduction needs substantial language & organization revision. The authors use "this/these" in many sentences, making hard to follow what exactly being referred in each sentence. Some examples are "These efforts …", "This is similar …", "These artifacts …". Furthermore, the introduction lacks a brief description of the existing methods mentioned by the authors (refs 8-14) as well as other relevant R packages such as EHR, rEHR, cleanEHR."

Regarding the Reviewer's first comment, we would like to note that the manuscript was revised by a professional proofreading service in American English. To address the Reviewer’s particular issues, however, we performed another round of editing on this revised version. Special attention has been given to the pronouns (where we originally

aimed to reduce the number of words). We have substituted the specific target nouns.

Regarding the Reviewer's suggestion of a brief description of other relevant R packages, we have inserted a brief section, where EHR and rEHR are mentioned. Since the cleanEHR package is no longer available in CRAN, we did not include it in our existing method description. We also added to the description some packages focused on time-series analysis, that although not focused on EHR data, could help in the detection of dataset shifts in EHR. The new paragraph (last paragraph in Background) reads as follows:

'In the R programming language, there are distinct packages that can help in managing or describing EHRs. For example, the rEHR package focuses on querying and filtering, while the EHR and comoRbidity packages allow the performance of descriptive, PheWAS, and comorbidity analyses.[24-26] Other packages, such as MTS or qcc, allow the performance of time-series or SPC analyses, which assist in detecting dataset shifts in EHRs.[27,28]

To our knowledge EHRtemporalVariability is the first package which provides specific dataset shift delineation, which can be used on raw EHRs and other data sources. The key advantage is its suitability for multi-modal and highly coded information, common features of biomedical data.'

Regarding the brief description of our previous methods (references 6, 9 and 11), we included those in the Methods section (second and third paragraph). Given the "Technical Note" style of this manuscript, we decided to place that information in Methods, as it would guide the consequent description of software functionalities.

R2Q2) "In the introduction the manuscript refers to existing methods based on statistical process control and time-series analysis but does not provide the references for these studies. It would be crucial to also add a short text on the contribution of this study (the proposed package) and how it relates / differs from the existing work to position the contribution of the study."

We thank the Reviewer for this suggestion. In response, we have first included the additional references to existing methods based on SPC and time-series (new references 18-23). We have also added the proposed short texts in the package CRAN and GitHub descriptions to better position the contribution of the study.

New CRAN description:
'Functions to delineate temporal dataset shifts in Electronic Health Records through the projection and visualization of dissimilarities among data temporal batches. This is done through the estimation of data statistical distributions over time and their projection in non-parametric statistical manifolds, uncovering the patterns of the data latent temporal variability. EHRtemporalVariability is particularly suited to multi-modal data and categorical variables with a high number of values, common features of biomedical data where traditional statistical process control or time-series methods may not be suitable. Dataset shifts can be explored and identified through visual analytics formats such as Data Temporal heatmaps and Information Geometric Temporal (IGT) plots. An additional 'EHRtemporalVariability' Shiny app can be used to load and explore the package results and even to allow the use of these functions to those users non-experienced in R coding. Preprint published in medRxiv (Sáez et al. 2020) <doi:10.1101/2020.04.07.20056564>.'

New GitHub description:
Please see https://github.com/hms-dbmi/EHRtemporalVariability#background

R2Q3) "In most case studies, the authors suggest that there was no apparent cause for the shift. It would certainly be useful to assign a confidence/statistical score (such as p-value/confidence interval) regarding the extremeness of observing the shit in concern. Moreover, as far as I understand the projection involves data compression due to the choice of the 2-3 dimensions to be displayed, it would also be useful to know the percentage of the information the visually presented plot captures (e.g., analogous to variant explained in a PCA plot)."

Powered by Editorial Manager® and ProduXion Manager® from Aries Systems Corporation

Although in the Mortality case study, the causes of the shifts are clearly determined (we refer the Reviewer to our previous paper in JAMIA), we found it more challenging to determine the exact cause in the BCH-ASD and NHDS cases. We did, however, discuss some possibilities of causes that are related to changes/updates in coding practices.

We concede the Reviewer's valid point in terms of the confidence/statistical score aspect. We have discussed the issue many times. In the end, we argue that the practical implementation of p-value scoring can be hindered by sample sizes (a tiny difference can be statistically significant in a large dataset) and number of categorical values of the datasets at hand. Specifically, categorical variables, such as those using ICD-9 or PheWAS codes, can have thousands of values, limiting approximations such as using Chi Square Goodness of Fit tests. Therefore, we recommend that users perform statistical tests at their discretion.

In response to the Reviewer's comment, we also conducted an additional experiment (attached) for the NHDS case study, using a Chi-Square Goodness of Fit test. We specifically sought statistical differences in the "diagcode1-phewascode" variable (principal diagnosis at discharge), which in 2008 split the NHDS dataset into two main subgroups. We found that the distribution in subgroup >=2008 was significantly different from the reference distribution in subgroup <2008 (p-value < 2.2e-16).

We also propose another approach: checking the extremeness of the probability distribution distances rather than the data. We refer the Reviewer to our previous work in DAMI, where a probability distribution Statistical Process Control method was provided for a bach-by-batch extremeness control.

Another option would be a bootstrap procedure that involves simulating Jensen Shannon Distances (JSDs) between two pre and post random bootstrap samples taken from the complete dataset (H0). The approach might produce a bootstrap distribution of JSD, upon which the JSD between the real pre vs post could be tested for likelihood. We argue, however, that this approach would fall out of the scope of this work.

Regarding the Reviewer's second comment, we agree that compression arises when using Multi-Dimensional Scaling (MDS) on the dissimilarity matrix between the batched statistical distributions (e.g, distributions of batches at a monthly basis). The percentage of captured information varies according to the complexity of the latent statistical manifold, as well as the number of variable category values or bins. There is also variation based on whether we use classical or non-metric MDS. Therefore, based on the Reviewer's comment, we have included this information as an additional attribute in the IGTprojection class resultant from the estimation of IGT plots on the selected number of dimensions. We note that in previous experiments with non-metric MDS, the resultant "Stress" was very close to 0, but classical MDS helped to visualize the large components of change, although with more loss of information (in R cmdscale measured as Goodness of Fit).

While the previous version of EHRtemporalVariability used internally classical MDS, we now have added the possibility of choosing between classical and non-metric MDS when estimating the IGT projections, where the resultant loss of information is returned in both cases. We thank the Reviewer for the suggestion.

R2Q4) "It seems the case studies mostly focus only on one of the 4 different types of changes which is somewhat the most trivial to identify (abrupt changes). I feel it would be beneficial to include/modify case studies on detecting trends (e.g., certain type of anti-depressant / painkiller use) and seasonality (e.g., flu) for demonstrating the full capability of the package / app. I think this is also important for educating users on how to interpret the IGT plots (i.e. spot different types of patterns)."

We agree with the Reviewer that it is important to educate users on how to interpret the IGT plots. In the manuscript, we focused predominantly on abrupt changes but also described others in the three case studies, as follows: ASD-BCH case study: Figure 2 caption: "Overall, there is a trend in the distribution changes across all the entire time frame."Mortality case study: Figure 3 highlights all four types of changes, please see

arrows labeled “a” (abrupt change), “b” (trend) and “c” (seasonality), while the temporal subgroups are defined by the split provided by a. (Note: we now have also highlighted outlying months due to acute flu epidemics). NHDS: the dedicated paragraph summarizes the changes, which are fully described in our previous work.

In response to the Reviewer’s suggestion, we have made two additional changes. First, we have added a new section in the package Vignette related to the interpretation of temporal changes in IGT projections (http://personales.upv.es/carsaesi/EHRtemporalVariability/EHRtemporalVariability.html#interpretation-of-temporal-changes-in-igt-projections). This new section describes the different types of changes as shown by IGT projections and then shows an example using the NHDS “Diagnosis code #1 PheWAS code” variable, where the four types of changes can be ascertained. In addition, we have emphasized throughout the manuscript the relationship between our findings and the four types of changes. We were most specific in the descriptions of case study results and also referenced the new content in the Vignette.

R2Q5) "In the conclusion I would be curious to see a brief discussion/recommendation on how one could address the dataset shifts in the data sets, specially in machine learning based on their expertise. For instance, would splitting the data into separate data sets in the model training such that the data before and after the data shift used to generate two models specific to certain time period be a plausible strategy? Or would they recommend to map (e.g., ICD9 to ICD10) / renormalize the data or perhaps add an additional feature pointing to the individual subgroups (e.g., before / after shift)."

We welcome the interest of the Reviewer in our recommendation of how to address dataset shifts. There is not a definitive answer, given that a general recommendation depends on the target data use. i.e., for a predictive model, one may think that ignoring past data before an abrupt change is a good option, since the latest data would most closely resemble future data. In fact, incremental learning approaches rely on this concept, where “forgetting mechanisms” can be introduced to regulate the manner in which past data fades from the model memory. Since we cannot predict that nature of new unobserved data, that is a well-supported hypothesis. It is, therefore, within our current investigation to include an additional feature that points to individuals subgroups, as the reviewer suggested. The feature would be equivalent to using mixed-effect models, but including the temporal factor.

We considered the suggestion of performing separate analyses for descriptive analyses, (or at least to report both the global and the separate results).

Regarding the mapping/renormalization, the mapping processes themselves can introduce temporal variability, given the possible distinct groups of terms, such as in the cases of ICD-9 yearly updates reported in the manuscript. We have included the following text in the last paragraph of results and discussion:

'In light of the changes uncovered by EHRtemporalVariability, we argue that users of the package can more accurately repurpose their data analyses. For example, in the presence of abrupt changes, one can compare the performance of predictive modelling using only the most recent temporal subgroups versus full data inclusion. The superiority of the former is supported by the hypothesis that newly observed data will more closely resemble the latest data.

In addition, incremental learning approaches can also be adopted to deal with abrupt changes and continuous trends in machine learning, as can introducing seasonal or subgroup-related effects on models. Finally, in cases of descriptive analyses, such as those in PheWAS studies, we suggest evaluating the possible effect of temporal changes in results by making separate analyses at distinct temporal subgroups, as opposed to performing more global analysis.'

We also refer the Reviewer to Table 4 in our previous work in JAMIA (https://doi.org/10.1093/jamia/ocw010) and Table 1 in our recent work in BMJ Open (http://dx.doi.org/10.1136/bmjopen-2019-034396), where specific recommendations are provided for different types of changes.

R2Q6) "On a technical note, what are the computational complexity for running the package (e.g., expected runtime on the used system configuration)? is there a limit on how many records / variables can be uploaded? It would also be great to know the size (in terms of MB / GB) of the benchmark data sets and the time it takes to upload / analyze the data in the Shiny app (in addition to the number of features and patients)."

We refer the Reviewer to the Supplementary Material, Section 3 “Performance measures,” in which most of these issues are discussed. Regarding the limits on the Shiny app, these will theoretically depend on the web server and hardware configuration of the system and on the specific size limits set on the Shiny app. These limits can be modified by the users in the Shiny app code, now set to 100MB (shiny.maxRequestSize=100*1024^2).

R2Q7) "The examples provided in the R package seem to work without major issues. The only error I got was in the following
> class(igtProj[[1]])
Error in igtProj[[1]] : this S4 class is not subsettable"

We thank the editor for pointing out the error. That was a typo, where igtProj[[1]] should be the list igtProjs[[1]]. We fixed it accordingly.

R2Q8) "Minor:
- can *by* constructed => be"

Changed accordingly.

"- to circumvent these issues => Is this actually circumventing or identifying? If the package does not do a correction it would be rather the latter, i.e. identifying the problem rather than correcting it."

Changed to: “to identify these temporal variability issues.”

"- highly coded information => Unclear what it refers to"

We have now introduced the term in the paragraph just before:

However, these approaches tend to promulgate loss of information, especially when deployed when using highly coded data, i.e., categorical variables with a particularly high number of values.

"- concern about complex variable processing => Again unclear"

Changed to:
Analyses can proceed using both the R package and Shiny app with minimum effort. Data can flow through the pipeline from its initial raw, individual-level state to the final results.

"- over 5 years => previously"

Changed accordingly.

"- machine-learning => machine learning"

Changed accordingly.

Additional Information:	
**Question**	**Response**
Are you submitting this manuscript to a special series or article collection?	No
Experimental design and statistics	Yes

Powered by Editorial Manager® and ProduXion Manager® from Aries Systems Corporation

Full details of the experimental design and statistical methods used should be given in the Methods section, as detailed in our Minimum Standards Reporting Checklist. Information essential to interpreting the data presented should be made available in the figure legends.<br><br>Have you included all the information requested in your manuscript?	
**Resources**<br><br>A description of all resources used, including antibodies, cell lines, animals and software tools, with enough information to allow them to be uniquely identified, should be included in the Methods section. Authors are strongly encouraged to cite Research Resource Identifiers (RRIDs) for antibodies, model organisms and tools, where possible.<br><br>Have you included the information requested as detailed in our Minimum Standards Reporting Checklist?	Yes
**Availability of data and materials**<br><br>All datasets and code on which the conclusions of the paper rely must be either included in your submission or deposited in publicly available repositories (where available and ethically appropriate), referencing such data using a unique identifier in the references and in the "Availability of Data and Materials" section of your manuscript.<br><br>Have you have met the above requirement as detailed in our Minimum Standards Reporting Checklist?	No
If not, please give reasons for any omissions below.	The software presented in the submitted Technical Note is Open Source, its code and documentation is fully available through GitHub and CRAN and all links are included in the manuscript. The paper is showcased with three case studies. The reproducibility of

as follow-up to "**Availability of data and materials**<br><br>All datasets and code on which the conclusions of the paper rely must be either included in your submission or deposited in publicly available repositories (where available and ethically appropriate), referencing such data using a unique identifier in the references and in the "Availability of Data and Materials" section of your manuscript.<br><br>Have you have met the above requirement as detailed in our Minimum Standards Reporting Checklist?<br><br>"	the evaluation of the tool as presented can be achieved using the publicly available NHDS dataset which, additionaly, is proxied within the package, included in its code examples, vignette, and in the on-line demo. Access to data of the other two case studies is restricted by their owners, where specific approval are required. We believe the conclusions raised by the paper of using our software can be fully reproduced using the publicly available NHDS dataset as described in the paper and documentation.

Manuscript

Click here to access/download;Manuscript;2020_ehrtemporalvariability_sae

EHRtemporalVariability: delineating temporal dataset shifts in electronic health records

Carlos Sáez,[1,2,*] Alba Gutiérrez-Sacristán,[2] Isaac Kohane,[2] Juan M García-Gómez,[1,‡] Paul Avillach[2,‡]

[1] Biomedical Data Science Lab, Instituto Universitario de Tecnologías de la Información y Comunicaciones (ITACA), Universitat Politècnica de València (UPV), Camino de Vera s/n, Valencia 46022, España

[2] Department of Biomedical Informatics, Harvard Medical School, Boston, MA, USA

* Corresponding author
‡Both to be regarded as senior authors

ORCID IDs:

Carlos Sáez: 0000-0002-9125-9856; Alba Gutiérrez-Sacristán: 0000-0002-1245-198X; Isaac Kohane: 0000-0003-2192-5160; Juan M Garcia-Gomez: 0000-0002-3851-1557; Paul Avillach: 0000-0002-0235-7543

Abstract

Background

Temporal variability in healthcare processes or protocols is intrinsic to medicine. Such variability can potentially introduce dataset shifts, a data quality issue when reusing electronic health records (EHRs) for secondary purposes. Temporal dataset shifts can present as trends, as well as abrupt or seasonal changes in the statistical distributions of data over time. The latter are particularly complicated to address in multi-modal and highly coded data. These changes, if not delineated, can harm population and data-driven research, such as machine learning. Given that biomedical research repositories are increasingly being populated with large historical data from EHRs, there is a need for specific software methods to help delineate temporal dataset shifts to ensure reliable data reuse.

Findings

EHRtemporalVariability is an Open Source R-package and Shiny-app designed to explore and identify temporal dataset shifts. EHRtemporalVariability estimates the statistical distributions

of coded and numerical data over time; projects their temporal-evolution through non-parametric Information Geometric Temporal plots; and enables the exploration of changes in variables through Data Temporal Heatmaps. We demonstrate the capability of EHRtemporalVariability to delineate dataset shifts in three impact case studies, one of them available for reproducibility.

Conclusions

EHRtemporalVariability enables the exploration and identification of dataset shifts, contributing to the broad examination and repurposing of large, longitudinal datasets. Our goal is to help ensure reliable data reuse for a wide range of biomedical data users. EHRtemporalVariability is designed for technical users who are programmatically utilizing the R-package, as well as users who are not familiar with programming via the Shiny user interface.

Availability: https://github.com/hms-dbmi/EHRtemporalVariability/

Reproducible vignette:

https://cran.rproject.org/web/packages/EHRtemporalVariability/vignettes/EHRtemporalVariability.html

On-line demo: http://ehrtemporalvariability.upv.es/

Keywords

Dataset shifts, data quality, temporal variability, scientific datasets, electronic health records, claims data, research repositories, information geometry, visual analytics, R package.

Background

The widespread adoption of data-sharing technologies, health information standards, and open-data initiatives are inspiring the creation of research data repositories that contain large-scale historical data from Electronic Health Records (EHRs).[1] These repositories represent a new class of longitudinal, real-world data (RWD), defined as large datasets collected over time from sources outside of clinical trials or specific research cohorts. While reuse of this data, ranging from clinical observations to molecular information, has begun to enhance the efficacy and generalization of biomedical and clinical research, efforts towards an efficient and reliable reuse of RWD are still in early stages.[2,3]

Most recently researchers from the machine learning community have identified EHR data as one of the most important sources of labeled data with which diagnostic and prognostic models can by constructed.[4] One of the major hurdles in reusing such EHR data, however, is its temporal variability. Indeed, clinical care processes and their local variations are permeated with a variety of batch effects and biases.[5–9] This situation is similar to that in genomics and other "omics" research, where batch effects can be introduced by technical sources of variation that have been added to samples during acquisition handling.[15,16]

Temporal variability artifacts—in form of dataset shifts—can impact data quality and challenge the secondary use of data, particularly for population and data-driven research,[8,10–12] as well as machine learning.[13,14] In addition, the EHR's themselves can contribute to variability, as they reflect the evolution of administrative practice and reimbursement policies, all of which can gradually or abruptly shift over time. For example, updates in coding systems, such as the International Classification of Diseases (ICD),[17] or modifications to clinical guidelines often lead to variable data representations across multiple diseases over time.

To circumvent these issues, researchers have traditionally deployed Statistical Process Control-based (SPC) methods, which expose the timepoints when reference changes had

occurred. Shewhart and Levey-Jennings charts, for example, have been employed in laboratory quality control efforts.[18,19] Similarly, autocorrelation or time-series-based approaches have been used to uncover periodicity and changes within summary statistics derived from longitudinal samples, such as batched averages.[20-23] When the dates of such reference changes are exposed, statistical tests can uncover significant differences between time periods. However, these approaches tend to promulgate loss of information, especially when deployed when using highly coded data, i.e., categorical variables with a particularly high number of values—such as using the ICD Ninth Revision, Clinical Modification (ICD-9-CM), which has over 16,000 distinct codes, as well as in multimodal statistical distributions, in which multiple sub-phenotypes are present.

In the R programming language, there are distinct packages that can help in managing or describing EHRs. For example, the *rEHR* package focuses on querying and filtering, while the *EHR* and *comoRbidity* packages allow the performance of descriptive, *PheWAS,* and comorbidity analyses.[24-26] Other packages, such as *MTS* or *qcc*, allow the performance of time-series or SPC analyses, which assist in detecting dataset shifts in EHRs.[27,28]

To our knowledge EHRtemporalVariability is the first package which provides specific dataset shift delineation, which can be used on raw EHRs and other data sources. The key advantage is its suitability for multi-modal and highly coded information, common features of biomedical data.

Methods

EHRtemporalVariability is designed to explore and identify the temporal variability of categorical and numerical data over time. The app provides the means to visually and analytically delineate dataset shifts in multi-modal and highly coded information. A key advantage is that no distributional assumptions are made. This enables straightforward use, as well as visual analytics on large EHR-coded and numerical variables with no loss of

information. In addition, the tool's methodological and iterative use can identify and define reference changes that might otherwise impede further research. Analyses can proceed using both the R package (RRID:SCR_001905) and Shiny app (RRID:SCR_001626) with minimum effort. Data can flow through the pipeline from its initial raw, individual-level state to the final results.

EHRtemporalVariability is based on the probabilistic temporal variability methods that we developed and validated previously,[6,9,11] namely Information-Geometric-Temporal (IGT) plots and Data Temporal Heatmaps (DTHs). We offer these for the first time as an open-source R package and Shiny app. Our method is based upon the estimation and comparison of data statistical distributions over time. (See online Methods.) IGT plots project time batches as a series of points. The distances between them correspond to the dissimilarity of their statistical distributions. This yields an empirical layout of temporal relationships between batches, namely a non-parametric temporal statistical manifold.

IGT plots allow users to visually identify four types of changes: 1) trends, represented as continuously flowing time batches; 2) abrupt changes, shown as gaps between groups of batches; 3) temporal subgroups, depicted as clusters of batches; and 4) seasonality, portrayed as temporal cycles. Batches are labeled by date and color-coded to distinguish seasonal effects. Additionally, IGT plots can include a smoothed trajectory of the information evolution over time. The IGT plot data also provides the means to identify those changes in order to model seasonal effects or apply clustering methods to depict temporal subgroups.[9] Complementing the IGT plots, DTHs allow users to explore changes in absolute and relative frequencies over time—and at multiple variable values, simultaneously (e.g., frequencies of phenotypes).

Overall, the EHRtemporalVariability R package (**Figure 1, left**) and Shiny app (**Figure 1, right**) provide a set of functionalities that allow users to perform three actions: loading and

processing datasets; running batched data analyses for estimation of DTHs and IGT projections; and visualizing these data through interactive plots. The R package also enables users to conduct these tasks programmatically, enabling more flexibility in data processing and further analysis of the resultant objects and embedding matrices.

The Shiny app provides a graphical user interface with two objectives. First, users unfamiliar with R programming can load *.csv* files and easily produce and visualize their results—which can be exported as a *.Rdata* file for further inspection in R. Second, we provide an exploratory, dynamic dashboard to improve the user experience, enabling a means to load results exported from the R package as a *.RData* file. We customized both the R package and Shiny app visualizations for users who are colorblind.

A more detailed description of methods is available in the Supplementary Material of the paper.

Figure 1. EHRtemporalVariability R package (left) and Shiny app (right) outline. The general workflow of the R package is organized as a set of functions for: (A1) data loading and preprocessing, (A2) data analysis, and (A3) data visualization. The main input is an R *data.frame*, in which one column defines the reference date. The classes of the remaining columns determine the variable's treatment for distribution estimation and plotting during analysis and visualization. (See online Methods section.) Specifically, "factor" and "character" receive categorical treatment, while "numeric," "integer," and "date" receive numerical treatment. The DTH object estimation takes the input "data.frame" and analysis parameters. These include temporal granularity; predefined distribution support (a range of possible values or bins for each variable, auto-calculated from data by default); handling of missing batches; and the choice of whether to smooth distributions in numerical variables. The DTH can be trimmed by values and date range. The IGT projection estimation takes as input the DTH and the desired number of dimensions for embedding. The DTH can be plotted as a dynamic Plotly (RRID:SCR_013991) heatmap, in which the color of each cell indicates the frequency (relative or absolute) at a specific date batch (column) for the value of a variable (categorical and numerical integer) or range or values (numerical continuous). IGT plots can be visualized as either two or three-dimensional dynamic Plotly plots. The input for the Shiny app can be either an *.RData* object exported from the R package ($B1_A$) or a raw .csv input file ($B1_B$). The Shiny app provides an interactive dashboard (B2) for controlling

the visualization parameters of the programmatic R functions. This is done via reactive sliders, selection boxes, and buttons. These have a direct effect on heatmaps and IGT plots. Further, we include different color palettes suited for different types of colorblindness. For further information about all the EHRtemporalVariability functionality see: https://cran.r-project.org/web/packages/EHRtemporalVariability/vignettes/EHRtemporalVariability.html.

Results and discussion

We validated the functionality of EHRtemporalVariability using three case studies. The first involved the i2b2 Boston Children's Hospital Autism Spectrum Disorders cohort (BCH-ASD), including 12,000 patients (1.2M ICD-9-CM clinical observations), recorded from 1981 to 2016. This project was reviewed by Boston Children's Institutional Review Board.

In this cohort, the IGT plot uncovered five abrupt changes of reference (**Figure 2a**). The most obvious was in billing codes, for which frequencies changed in October 1998 (**Figure 2a-a$_2$**). Accordingly, we discovered an abrupt change in the relative frequencies of ICD-9-CM codes during that month. Specifically, the DTH of the ICD-9-CM codes (Supplementary Material Fig 1) showed an abrupt decrease in frequency of codes: 780 (general symptoms), 780.9 (other general symptoms) and 289.9 (unspecified diseases of blood and blood-forming organs). We also tracked increases in more specific 780.x codes; 296.x codes (episodic mood disorders), and other trends, represented as gradual changes.

While investigating the root cause of the October 1998 reference change, we found that it coincided with a yearly ICD-9-CM update. However, there was no apparent relationship between documented changes and our findings.

To further investigate this variability, we mapped ICD-9-CM to Phenome Wide Association Studies (PheWAS) codes.[29] We removed all the observations listed as "other symptoms" and "other tests." Still, the abrupt change persisted even when we delineated changes for further specific comorbidities (**Figures 2b, 2c**). Intriguingly, the absolute number of observations also

increased at the start of the month. Although this reference change appears to be motivated by a systemic or protocol change, the exact cause remains unclear. We suggest that this reference change is a potential dataset shift that should be considered in any future BCH-ASD data analysis.

Figure 2. Delineation of dataset shifts in the Boston Children's Hospital EHR Autism Spectrum Disorders historical clinical observations. (**a**) IGT plot describing the evolution of distributions of ICD-9-CM codes over time—specifically monthly time batches, taken from March 1989 to June 2016. The projection of time batches is based on embedding the dissimilarities among their distributions using multidimensional scaling. The IGT plot axis corresponds to the three first temporal components of variance. Several slight abrupt changes are apparent during October 1991 (a_1), January 2003 (a_3), and December 2011 (a_4). Major abrupt changes appear during October 1998 (a_2) and October 2015 (a_5). Overall, there is a trend in the distribution changes across all the entire time frame. Text labels are formatted as "yym," where "yy" is a two-digit year and "m" is an abbreviated month, as {'J', 'F', 'M', 'A', 'm', 'j', 'x', 'a', 'S', 'O', 'N', 'D'}. (**b**) Data Temporal Heatmap of the 20 most frequent relative frequencies of PheWAS codes text. (**c**) Data Temporal Heatmap of the 20 most frequent absolute frequencies of PheWAS codes text. The major driver for (a_2) was a decrease in "other symptoms" and "other tests" codes. Thus, we excluded these to investigate the effect on comorbidities and obtained (b) and (c). Changes in October 1998 (a_2) include increases in the frequencies of "constipation," "major depressive disorder," "symptoms involving digestive system," and "type 2 diabetes." Other minor decreases included "cystic fibrosis," "other diseases of blood and blood forming-organs," and "type 1 diabetes," among "others. As observed in (c), some of the delineated changes are time-correlated with alterations in absolute frequencies.

The second case study replicates a baseline experiment we previously performed using the Mortality Registry of Valencia, Spain.[11] The registry recorded 512,000 deaths between 2000 and 2012. Similar to the Boston Children's results, the registry's statistical distributions changed abruptly in 2009, following an update in the fields of the Spanish National Death Certificate. Notably, this reference change impacted the Basic Cause of Death, a variable used for reporting National and International death statistics (**Figure 3a,** and Supplementary Material Fig 2). This occurred even after the variable was retrospectively corrected. The results also showed an overall trend through the entire period of the study (Figure 3b), a yearly

seasonality of causes of death (Figure 3c), and spotted outlier months associated to flu epidemics in 2002, 2005 and 2009 (Figure 3 d_1, d_2 and d_3, respectively).

Figure 3. IGT plot of the Basic Cause of Death in the Mortality Registry of the Region of Valencia, Spain, coded with ICD Tenth Revision (ICD-10) Mortality Causes List 1. (**a**) The major abrupt change associated with the update of the National Certificate of Death is depicted as a dotted line that splits the main trend through the entire period of study (**b**), trend that lays in dimension D1. (**c**) Yearly seasonality of causes of death, highlighted by coloring scheme and trajectory cycles and laid out across dimension D2. (**d_1**, **d_2**, **d_3**) Flu epidemics in 2002, 2005 and 2009 as outlying batches and showing fast trajectory deviations. Text labels are formatted as"'yym," where "yy" is a two-digit year and "m" is an abbreviated month as {'J', 'F', 'M', 'A', 'm', 'j', 'x', 'a', 'S', 'O', 'N', 'D'}. The drivers for (a) included a relatively abrupt decrease in "symptoms, signs, and abnormal clinical and laboratory findings, not elsewhere classified" and an increase in "hypertensive diseases," among others.

Finally, we validated EHRtemporalVariability with the National Hospital Discharge Survey (NHDS), an open dataset that includes 3.25M inpatient discharges from US hospitals (2000-2010) and both demographic and ICD-9-CM-coded data. Again, we uncovered several abrupt changes throughout multiple variables, [6,9] including the re-coding of discharge age in 2008; ICD-9-CM diagnoses (**Figure 1**); procedure codes; and yearly abrupt changes in diagnosis-related group codes. These findings were in addition to the expected context-induced trends and seasonality. After mapping the NHDS ICD-9-CM to PheWAS codes, we noted that notable changes remained, including those appearing in October 2007, coincident with the yearly ICD-9-CM update. Note: this case study is available for replication within the package and Shiny app demonstration at http://ehrtemporalvariability.upv.es/, and a tutorial on how to interpret temporal changes in IGT plots using NHDS data is available in the package vignette. Performance measures for the three case studies are described in the Supplementary Material.

In light of the changes uncovered by EHRtemporalVariability, we argue that users of the package can more accurately repurpose their data analyses. For example, in the presence of

abrupt changes, one can compare the performance of predictive modelling using only the most recent temporal subgroups versus full data inclusion. The superiority of the former is supported by the hypothesis that newly observed data will more closely resemble the latest data.

In addition, incremental learning approaches can also be adopted to deal with abrupt changes and continuous trends in machine learning, as can introducing seasonal or subgroup-related effects on models. Finally, in cases of descriptive analyses, such as those in PheWAS studies, we suggest evaluating the possible effect of temporal changes in results by making separate analyses at distinct temporal subgroups, as opposed to performing more global analysis.

Conclusions

In conclusion, EHRtemporalVariability is a data quality assessment tool that enables the broad exploration and repurposing of large data sets collected over time. We view the app as a key steppingstone toward the identification of dataset shifts for data reuse, specifically in machine learning. Target users are biomedical data scientists and bioinformaticians, as well as epidemiologists and hospital data managers. The tool can assist in exploring the effects of system, protocol, and environment-induced changes on data. We also encourage the use of EHRtemporalVariability to analyze the impact of the adoption of new coding systems such as ICD-10.[30] EHRtemporalVariability can be used on any additional coded and numerical data modalities and, because it is open-source, the app can be extended with new functionality or uses by the scientific community.

Availability of code and resources

- Project name: EHRtemporalVariability
- Project home page: https://github.com/hms-dbmi/EHRtemporalVariability/
- Operating system(s): Platform independent
- Programming language: R

- Other requirements: R 3.3.0, dplyr, plotly, shiny, zoo, xts, lubridate, RColorBrewer, viridis, scales, methods, MASS
- License: Apache License 2.0
- CRAN repository: https://cran.r-project.org/package=EHRtemporalVariability
- bio.tools ID: biotools:ehrtemporalvariability
- SciCrunch ID: RRID:SCR_018663
- Shiny-app repository: https://github.com/hms-dbmi/EHRtemporalVariability-shiny
- Reproducible vignette: https://cran.r-project.org/web/packages/EHRtemporalVariability/vignettes/EHRtemporalVariability.html
- On-line Shiny app demo (for privacy reasons loading raw .csv data is disabled): http://ehrtemporalvariability.upv.es/

Data availability

The data of the NHDS case study is publicly available at https://www.cdc.gov/nchs/nhds/index.htm. A random subset of this dataset is available as a proxy for testing purposes within the EHRtemporalVariability package, and reproducible examples are available within the package help, its vignette, and the on-line demo. An archival snapshot of the code is available in the GigaScience GigaDB repository. [31] Access to BCH-ASD case study data is restricted by Boston's Children's Institutional Review Board. Access to the Mortality case study data is restricted by the Conselleria de Sanitat Universal i Salut Pública, Generalitat Valenciana, Spain.

Additional files

We provide a Supplementary material file including: (1) the technical details of the methods, (2) supplementary figures, and (3) a performance measures test.

Abbreviations

BCH-ASD: Boston Children's Hospital Autism Spectrum Disorders cohort; DTH: Data Temporal Heatmap; EHR: Electronic Health Record; ICD: International Classification of Diseases; ICD-9-CM: ICD Ninth Revision, Clinical Modification; IGT plot: Information Geometric Temporal plot; NHDS: National Hospital Discharge Survey; PheWAS: Phenome Wide Association Studies

Acknowledgments

This work was supported by UPV grant PAID-00-17, GVA grant BEST/2018, and projects H2020-SC1-2016-CNECT No. 727560 and H2020-SC1-BHC-2018-2020 No. 825750. The authors thank the community that collaboratively created the Open Source R software and packages used in this work. A special thanks to UpSetR, which inspired our Shiny wrapper landing page.

Competing Interests

The authors declare that they have no competing interests.

References

1. Gewin, V. Data sharing: An open mind on open data. *Nature* 529, 117–119 (2016).
2. Katzan, I. L. & Rudick, R. A. Time to integrate clinical and research informatics. *Sci. Transl. Med.* 4, 162fs41 (2012).
3. Zhu, L. & Zheng, W. J. Informatics, Data Science, and Artificial Intelligence. *JAMA* 320, 1103–1104 (2018).
4. Rajkomar, A., Dean, J. & Kohane, I. Machine Learning in Medicine. *N. Engl. J. Med.* 380, 1347–1358 (2019).
5. Andreu-Perez, J., Poon, C. C. Y., Merrifield, R. D., Wong, S. T. C. & Yang, G.-Z. Big data for health. *IEEE J Biomed Health Inform* 19, 1193–1208 (2015).
6. Sáez, C., Rodrigues, P. P., Gama, J., Robles, M. & García-Gómez, J. M. Probabilistic

change detection and visualization methods for the assessment of temporal stability in biomedical data quality. *Data Min. Knowl. Discov.* 29, 950–975 (2015).

7. Schlegel, D. R. & Ficheur, G. Secondary Use of Patient Data: Review of the Literature Published in 2016. *Yearb. Med. Inform.* 26, 68–71 (2017).
8. Agniel, D., Kohane, I. S. & Weber, G. M. Biases in electronic health record data due to processes within the healthcare system: retrospective observational study. *BMJ* 361, k1479 (2018).
9. Sáez, C. & García-Gómez, J. M. Kinematics of Big Biomedical Data to characterize temporal variability and seasonality of data repositories: Functional Data Analysis of data temporal evolution over non-parametric statistical manifolds. *Int. J. Med. Inform.* 119, 109–124 (2018).
10. Knight, L., Halech, R., Martin, C. & Mortimer, L. *Impact of changes in diabetes coding on Queensland hospital principal diagnosis morbidity data*. (Health Statistics Centre, Queensland Health, 2011). https://www.health.qld.gov.au/hsu/tech_report/techreport_9.pdf
11. Sáez, C. *et al.* Applying probabilistic temporal and multisite data quality control methods to a public health mortality registry in Spain: a systematic approach to quality control of repositories. *J. Am. Med. Inform. Assoc.* 23, 1085–1095 (2016).
12. Wright, A. *et al.* Best practices for preventing malfunctions in rule-based clinical decision support alerts and reminders: Results of a Delphi study. *Int. J. Med. Inform.* 118, 78–85 (2018).
13. Sugiyama, M., Lawrence, N. D., Schwaighofer, A. & Others. *Dataset shift in machine learning*. (The MIT Press, 2017). ISBN: 9780262170055
14. Moreno-Torres, J. G., Raeder, T., Alaiz-Rodríguez, R., Chawla, N. V. & Herrera, F. A unifying view on dataset shift in classification. *Pattern Recognit.* 45, 521–530 (2012).
15. Leek, J. T. *et al.* Tackling the widespread and critical impact of batch effects in high-throughput data. *Nat. Rev. Genet.* 11, 733–739 (2010).
16. Goh, W. W. B., Wang, W. & Wong, L. Why Batch Effects Matter in Omics Data, and

How to Avoid Them. *Trends Biotechnol.* 35, 498–507 (2017).

17. Centers for Disease Control and Prevention's (CDC), National Center for Health Statistics, U.S. Department of Health & Human Services. International Classification of Diseases,Ninth Revision, Clinical Modification (ICD-9-CM). (2015). https://www.cdc.gov/nchs/icd/icd9cm.htm. (Accessed: 16th July 2018)
18. Shewhart, W. A. & Deming. W. E. Statistical Method from the Viewpoint of Quality Control. New York, Dover (1986).
19. Westgard, J.O. Basic QC Practices: Training in Statistical Quality Control for Medical Laboratories. Madison, WI (2010). ISBN:9781886958074
20. Svolba, G. & Bauer, P. Statistical quality control in clinical trials. *Control. Clin. Trials* 20 (6) 519–530 (1999).
21. Bray, F. & Parkin, D. M. Evaluation of data quality in the cancer registry: principles and methods. Part I: comparability, validity and timeliness. *Eur. J. Cancer*, 45(5), 747-755 (2009).
22. Kahn, M. G., Raebel, M. A., Glanz, J. M., Riedlinger, K., & Steiner, J. F. A pragmatic framework for single-site and multisite data quality assessment in electronic health record-based clinical research. *Med. care.* 50 (2012).
23. Box, G.E., Jenkins, G.M., Reinsel, G.C. & Ljung, G.M. Time Series Analysis: Forecasting and Control, John Wiley & Sons (2015).
24. Springate, D.A., Parisi. R., Olier. I., Reeves, D., Kontopantelis, E. rEHR: An R package for manipulating and analysing Electronic Health Record data. PLoS ONE 12(2): e0171784 (2017).
25. Choi, L., Carroll, R. J., Beck, C., Mosley, J. D., Roden, D. M., Denny, J. C., & Van Driest, S. L. Evaluating statistical approaches to leverage large clinical datasets for uncovering therapeutic and adverse medication effects. Bioinformatics, 34(17), 2988-2996 (2018).
26. Gutiérrez-Sacristán, A., Bravo, À., Giannoula, A., Mayer, M. A., Sanz, F., & Furlong, L. I. comoRbidity: an R package for the systematic analysis of disease comorbidities.

Bioinformatics, 34(18), 3228-3230. (2018).

27. Tsay, R. S. Multivariate time series analysis: with R and financial applications. John Wiley & Sons. (2013).

28. Scrucca, L. qcc: an R package for quality control charting and statistical process control. R News 4/1, 11-17. (2004).

29. Denny, J. C. *et al.* Systematic comparison of phenome-wide association study of electronic medical record data and genome-wide association study data. *Nat. Biotechnol.* 31, 1102–1110 (2013).

30. Khera, R., Dorsey, K. B. & Krumholz, H. M. Transition to the ICD-10 in the United States: An Emerging Data Chasm. *JAMA* 320, 133–134 (2018).

31. Sáez C; Gutiérrez-Sacristán A; Kohane I; García-Gómez JM; Avillach P (2020): Supporting data for "EHRtemporalVariability: delineating temporal dataset shifts in electronic health records" GigaScience Database. http://dx.doi.org/10.5524/100766

Manuscript (highlighted changes) Click here to access/download;Manuscript;2020_ehrtemporalvariability_sae

EHRtemporalVariability: delineating temporal dataset shifts in electronic health records

Carlos Sáez,[1,2,*] Alba Gutiérrez-Sacristán,[2] Isaac Kohane,[2] Juan M García-Gómez,[1,†] Paul Avillach[2,†]

[1] Biomedical Data Science Lab, Instituto Universitario de Tecnologías de la Información y Comunicaciones (ITACA), Universitat Politècnica de València (UPV), Camino de Vera s/n, Valencia 46022, España

[2] Department of Biomedical Informatics, Harvard Medical School, Boston, MA, USA

* Corresponding author
† Both to be regarded as last authors

Abstract

Background

Temporal variability in healthcare processes or protocols is intrinsic to medicine. Such variability can potentially introduce dataset shifts, a data quality issue when reusing electronic health records (EHRs) for secondary purposes. Temporal dataset shifts can present as trends, as well as abrupt or seasonal changes in the statistical distributions of data over time. The latter are particularly complicated to address in multi-modal and highly coded data. These changes, if not delineated, can harm population and data-driven research, such as machine learning. Given that biomedical research repositories are increasingly being populated with large historical data from EHRs, there is a need for specific software methods to help delineate temporal dataset shifts to ensure reliable data reuse.

Findings

EHRtemporalVariability is an Open Source R-package and Shiny-app designed to explore and identify temporal dataset shifts. EHRtemporalVariability estimates the statistical distributions of coded and numerical data over time; projects their temporal-evolution through non-parametric Information Geometric Temporal plots; and enables the exploration of changes in variables through Data Temporal Heatmaps. We demonstrate the capability of EHRtemporalVariability to delineate dataset shifts in three impact case studies, one of them available for reproducibility.

Conclusions

EHRtemporalVariability enables the exploration and identification of dataset shifts, contributing to the broad examination and repurposing of large, longitudinal datasets. Our goal is to help ensure reliable data reuse for a wide range of biomedical data users. EHRtemporalVariability is designed for technical users who are programmatically utilizing the R-package, as well as users who are not familiar with programming via the Shiny user interface.

Availability: https://github.com/hms-dbmi/EHRtemporalVariability/

Reproducible vignette:

https://cran.rproject.org/web/packages/EHRtemporalVariability/vignettes/EHRtemporalVariability.html

On-line demo: http://ehrtemporalvariability.upv.es/

Keywords

Dataset shifts, data quality, temporal variability, scientific datasets, electronic health records, claims data, research repositories, information geometry, visual analytics, R package.

Background

The widespread adoption of data-sharing technologies, health information standards, and open-data initiatives are inspiring the creation of research data repositories that contain large-scale historical data from Electronic Health Records (EHRs).[1] These repositories represent a new class of longitudinal, real-world data (RWD), defined as large datasets collected over time from sources outside of clinical trials or specific research cohorts. While reuse of this data, ranging from clinical observations to molecular information, has begun to enhance the efficacy and generalization of biomedical and clinical research, efforts towards an efficient and reliable reuse of RWD are still in early stages.[2,3]

Most recently researchers from the machine learning community have identified EHR data as one of the most important sources of labeled data with which diagnostic and prognostic models can by constructed.[4] One of the major hurdles in reusing such EHR data, however, is its temporal variability. Indeed, clinical care processes and their local variations are permeated with a variety of batch effects and biases.[5–9] This situation is similar to that in genomics and other "omics" research, where batch effects can be introduced by technical sources of variation that have been added to samples during acquisition handling.[15,16]

Temporal variability artifacts—in form of dataset shifts—can impact data quality and challenge the secondary use of data, particularly for population and data-driven research,[8,10–12] as well as machine learning.[13,14] In addition, the EHR's themselves can contribute to variability, as they reflect the evolution of administrative practice and reimbursement policies, all of which can gradually or abruptly shift over time. For example, updates in coding systems, such as the International Classification of Diseases (ICD),[17] or modifications to clinical guidelines often lead to variable data representations across multiple diseases over time.

To circumvent these issues, researchers have traditionally deployed Statistical Process Control-based (SPC) methods, which expose the timepoints when reference changes had

occurred. Shewhart and Levey-Jennings charts, for example, have been employed in laboratory quality control efforts.[18,19] Similarly, autocorrelation or time-series-based approaches have been used to uncover periodicity and changes within summary statistics derived from longitudinal samples, such as batched averages.[20-23] When the dates of such reference changes are exposed, statistical tests can uncover significant differences between time periods. However, these approaches tend to promulgate loss of information, especially when deployed when using highly coded data, i.e., categorical variables with a particularly high number of values—such as using the ICD Ninth Revision, Clinical Modification (ICD-9-CM), which has over 16,000 distinct codes, as well as in multimodal statistical distributions, in which multiple sub-phenotypes are present.

In the R programming language, there are distinct packages that can help in managing or describing EHRs. For example, the *rEHR* package focuses on querying and filtering, while the *EHR* and *comoRbidity* packages allow the performance of descriptive, *PheWAS,* and comorbidity analyses.[24-26] Other packages, such as *MTS* or *qcc*, allow the performance of time-series or SPC analyses, which assist in detecting dataset shifts in EHRs.[27,28]

To our knowledge EHRtemporalVariability is the first package which provides specific dataset shift delineation, which can be used on raw EHRs and other data sources. The key advantage is its suitability for multi-modal and highly coded information, common features of biomedical data.

Methods

EHRtemporalVariability is designed to explore and identify the temporal variability of categorical and numerical data over time. The app provides the means to visually and analytically delineate dataset shifts in multi-modal and highly coded information. A key advantage is that no distributional assumptions are made. This enables straightforward use, as well as visual analytics on large EHR-coded and numerical variables with no loss of

information. In addition, the tool's methodological and iterative use can identify and define reference changes that might otherwise impede further research. Analyses can proceed using both the R package and Shiny app with minimum effort. Data can flow through the pipeline from its initial raw, individual-level state to the final results.

EHRtemporalVariability is based on the probabilistic temporal variability methods that we developed and validated previously,[6,9,11] namely Information-Geometric-Temporal (IGT) plots and Data Temporal Heatmaps (DTHs). We offer these for the first time as an open-source R package and Shiny app. Our method is based upon the estimation and comparison of data statistical distributions over time. (See online Methods.) IGT plots project time batches as a series of points. The distances between them correspond to the dissimilarity of their statistical distributions. This yields an empirical layout of temporal relationships between batches, namely a non-parametric temporal statistical manifold.

IGT plots allow users to visually identify four types of changes: 1) trends, represented as continuously flowing time batches; 2) abrupt changes, shown as gaps between groups of batches; 3) temporal subgroups, depicted as clusters of batches; and 4) seasonality, portrayed as temporal cycles. Batches are labeled by date and color-coded to distinguish seasonal effects. Additionally, IGT plots can include a smoothed trajectory of the information evolution over time. The IGT plot data also provides the means to identify those changes in order to model seasonal effects or apply clustering methods to depict temporal subgroups.[9] Complementing the IGT plots, DTHs allow users to explore changes in absolute and relative frequencies over time—and at multiple variable values, simultaneously (e.g., frequencies of phenotypes).

Overall, the EHRtemporalVariability R package (**Figure 1, left**) and Shiny app (**Figure 1, right**) provide a set of functionalities that allow users to perform three actions: loading and processing datasets; running batched data analyses for estimation of DTHs and IGT

projections; and visualizing these data through interactive plots. The R package also enables users to conduct these tasks programmatically, enabling more flexibility in data processing and further analysis of the resultant objects and embedding matrices.

The Shiny app provides a graphical user interface with two objectives. First, users unfamiliar with R programming can load *.csv* files and easily produce and visualize their results—which can be exported as a *.Rdata* file for further inspection in R. Second, we provide an exploratory, dynamic dashboard to improve the user experience, enabling a means to load results exported from the R package as a *.RData* file. We customized both the R package and Shiny app visualizations for users who are colorblind.

A more detailed description of methods is available in the Supplementary Material of the paper.

Figure 1. EHRtemporalVariability R package (left) and Shiny app (right) outline. The general workflow of the R package is organized as a set of functions for: (A1) data loading and preprocessing, (A2) data analysis, and (A3) data visualization. The main input is an R *data.frame*, in which one column defines the reference date. The classes of the remaining columns determine the variable's treatment for distribution estimation and plotting during analysis and visualization. (See online Methods section.) Specifically, "factor" and "character*"* receive categorical treatment, while "numeric," "integer,*"* and "date" receive numerical treatment. The DTH object estimation takes the input "data.frame*"* and analysis parameters. These include temporal granularity; predefined distribution support (a range of possible values or bins for each variable, auto-calculated from data by default); handling of missing batches; and the choice of whether to smooth distributions in numerical variables. The DTH can be trimmed by values and date range. The IGT projection estimation takes as input the DTH and the desired number of dimensions for embedding. The DTH can be plotted as a dynamic Plotly heatmap, in which the color of each cell indicates the frequency (relative or absolute) at a specific date batch (column) for the value of a variable (categorical and numerical integer) or range or values (numerical continuous). IGT plots can be visualized as either two or three-dimensional dynamic Plotly plots. The input for the Shiny app can be either an *.RData* object exported from the R package ($B1_A$) or a raw .csv input file ($B1_B$). The Shiny app provides an interactive dashboard (B2) for controlling the visualization

parameters of the programmatic R functions. This is done via reactive sliders, selection boxes, and buttons. These have a direct effect on heatmaps and IGT plots. Further, we include different color palettes suited for different types of colorblindness. For further information about all the EHRtemporalVariability functionality see: https://cran.r-project.org/web/packages/EHRtemporalVariability/vignettes/EHRtemporalVariability.html.

Results and discussion

We validated the functionality of EHRtemporalVariability using three case studies. The first involved the i2b2 Boston Children's Hospital Autism Spectrum Disorders cohort (BCH-ASD), including 12,000 patients (1.2M ICD-9-CM clinical observations), recorded from 1981 to 2016. This project was reviewed by Boston Children's Institutional Review Board.

In this cohort, the IGT plot uncovered five abrupt changes of reference (**Figure 2a**). The most obvious was in billing codes, for which frequencies changed in October 1998 (**Figure 2a-a$_2$**). Accordingly, we discovered an abrupt change in the relative frequencies of ICD-9-CM codes during that month. Specifically, the DTH of the ICD-9-CM codes (Supplementary Material Fig 1) showed an abrupt decrease in frequency of codes: 780 (general symptoms), 780.9 (other general symptoms) and 289.9 (unspecified diseases of blood and blood-forming organs). We also tracked increases in more specific 780.x codes; 296.x codes (episodic mood disorders), and other trends, represented as gradual changes.

While investigating the root cause of the October 1998 reference change, we found that it coincided with a yearly ICD-9-CM update. However, there was no apparent relationship between documented changes and our findings.

To further investigate this variability, we mapped ICD-9-CM to Phenome Wide Association Studies (PheWAS) codes.[29] We removed all the observations listed as "other symptoms" and "other tests." Still, the abrupt change persisted even when we delineated changes for further specific comorbidities (**Figures 2b, 2c**). Intriguingly, the absolute number of observations also

increased at the start of the month. Although this reference change appears to be motivated by a systemic or protocol change, the exact cause remains unclear. We suggest that this reference change is a potential dataset shift that should be considered in any future BCH-ASD data analysis.

Figure 2. Delineation of dataset shifts in the Boston Children's Hospital EHR Autism Spectrum Disorders historical clinical observations. (**a**) IGT plot describing the evolution of distributions of ICD-9-CM codes over time—specifically monthly time batches, taken from March 1989 to June 2016. The projection of time batches is based on embedding the dissimilarities among their distributions using multidimensional scaling. The IGT plot axis corresponds to the three first temporal components of variance. Several slight abrupt changes are apparent during October 1991 (a_1), January 2003 (a_3), and December 2011 (a_4). Major abrupt changes appear during October 1998 (a_2) and October 2015 (a_5). Overall, there is a trend in the distribution changes across all the entire time frame. Text labels are formatted as "yym," where "yy" is a two-digit year and "m" is an abbreviated month, as {'J', 'F', 'M', 'A', 'm', 'j', 'x', 'a', 'S', 'O', 'N', 'D'}. (**b**) Data Temporal Heatmap of the 20 most frequent relative frequencies of PheWAS codes text. (**c**) Data Temporal Heatmap of the 20 most frequent absolute frequencies of PheWAS codes text. The major driver for (a_2) was a decrease in "other symptoms" and "other tests" codes. Thus, we excluded these to investigate the effect on comorbidities and obtained (b) and (c). Changes in October 1998 (a_2) include increases in the frequencies of "constipation," "major depressive disorder," "symptoms involving digestive system," and "type 2 diabetes." Other minor decreases included "cystic fibrosis," "other diseases of blood and blood forming-organs," and "type 1 diabetes," among "others. As observed in (c), some of the delineated changes are time-correlated with alterations in absolute frequencies.

The second case study replicates a baseline experiment we previously performed using the Mortality Registry of Valencia, Spain.[11] The registry recorded 512,000 deaths between 2000 and 2012. Similar to the Boston Children's results, the registry's statistical distributions changed abruptly in 2009, following an update in the fields of the Spanish National Death Certificate. Notably, this reference change impacted the Basic Cause of Death, a variable used for reporting National and International death statistics (**Figure 3a,** and Supplementary Material Fig 2). This occurred even after the variable was retrospectively corrected. The results also showed an overall trend through the entire period of the study (Figure 3b), a yearly

seasonality of causes of death (Figure 3c), and spotted outlier months associated to flu epidemics in 2002, 2005 and 2009 (Figure 3 d_1, d_2 and d_3, respectively).

Figure 3. IGT plot of the Basic Cause of Death in the Mortality Registry of the Region of Valencia, Spain, coded with ICD Tenth Revision (ICD-10) Mortality Causes List 1. (**a**) The major abrupt change associated with the update of the National Certificate of Death is depicted as a dotted line that splits the main trend through the entire period of study (**b**), trend that lays in dimension D1. (**c**) Yearly seasonality of causes of death, highlighted by coloring scheme and trajectory cycles and laid out across dimension D2. (**d_1, d_2, d_3**) Flu epidemics in 2002, 2005 and 2009 as outlying batches and showing fast trajectory deviations. Text labels are formatted as"'yym," where "yy" is a two-digit year and "m" is an abbreviated month as {'J', 'F', 'M', 'A', 'm', 'j', 'x', 'a', 'S', 'O', 'N', 'D'}. The drivers for (a) included a relatively abrupt decrease in "symptoms, signs, and abnormal clinical and laboratory findings, not elsewhere classified" and an increase in "hypertensive diseases," among others.

Finally, we validated EHRtemporalVariability with the National Hospital Discharge Survey (NHDS), an open dataset that includes 3.25M inpatient discharges from US hospitals (2000-2010) and both demographic and ICD-9-CM-coded data. Again, we uncovered several abrupt changes throughout multiple variables, [6,9] including the re-coding of discharge age in 2008; ICD-9-CM diagnoses (**Figure 1**); procedure codes; and yearly abrupt changes in diagnosis-related group codes. These findings were in addition to the expected context-induced trends and seasonality. After mapping the NHDS ICD-9-CM to PheWAS codes, we noted that notable changes remained, including those appearing in October 2007, coincident with the yearly ICD-9-CM update. Note: this case study is available for replication within the package and Shiny app demonstration at http://ehrtemporalvariability.upv.es/, and a tutorial on how to interpret temporal changes in IGT plots using NHDS data is available in the package vignette. Performance measures for the three case studies are described in the Supplementary Material.

In light of the changes uncovered by EHRtemporalVariability, we argue that users of the package can more accurately repurpose their data analyses. For example, in the presence of

abrupt changes, one can compare the performance of predictive modelling using only the most recent temporal subgroups versus full data inclusion. The superiority of the former is supported by the hypothesis that newly observed data will more closely resemble the latest data.

In addition, incremental learning approaches can also be adopted to deal with abrupt changes and continuous trends in machine learning, as can introducing seasonal or subgroup-related effects on models. Finally, in cases of descriptive analyses, such as those in PheWAS studies, we suggest evaluating the possible effect of temporal changes in results by making separate analyses at distinct temporal subgroups, as opposed to performing more global analysis.

Conclusions

In conclusion, EHRtemporalVariability is a data quality assessment tool that enables the broad exploration and repurposing of large data sets collected over time. We view the app as a key steppingstone toward the identification of dataset shifts for data reuse, specifically in machine learning. Target users are biomedical data scientists and bioinformaticians, as well as epidemiologists and hospital data managers. The tool can assist in exploring the effects of system, protocol, and environment-induced changes on data. We also encourage the use of EHRtemporalVariability to analyze the impact of the adoption of new coding systems such as ICD-10.[30] EHRtemporalVariability can be used on any additional coded and numerical data modalities and, because it is open-source, the app can be extended with new functionality or uses by the scientific community.

Availability of code and resources

- Project name: EHRtemporalVariability
- Project home page: https://github.com/hms-dbmi/EHRtemporalVariability/
- Operating system(s): Platform independent
- Programming language: R

- Other requirements: R 3.3.0, dplyr, plotly, shiny, zoo, xts, lubridate, RColorBrewer, viridis, scales, methods, MASS
- License: Apache License 2.0
- CRAN repository: https://cran.r-project.org/package=EHRtemporalVariability
- bio.tools ID: biotools:ehrtemporalvariability
- SciCrunch ID: RRID:SCR_018663
- Shiny-app repository: https://github.com/hms-dbmi/EHRtemporalVariability-shiny
- Reproducible vignette: https://cran.r-project.org/web/packages/EHRtemporalVariability/vignettes/EHRtemporalVariability.html
- On-line Shiny app demo (for privacy reasons loading raw .csv data is disabled): http://ehrtemporalvariability.upv.es/

Data availability

The data of the NHDS case study is publicly available at https://www.cdc.gov/nchs/nhds/index.htm. A random subset of this dataset is available as a proxy for testing purposes within the EHRtemporalVariability package, and reproducible examples are available within the package help, its vignette, and the on-line demo. Access to BCH-ASD case study data is restricted by Boston's Children's Institutional Review Board. Access to the Mortality case study data is restricted by the Conselleria de Sanitat Universal i Salut Pública, Generalitat Valenciana, Spain.

Additional files

We provide a Supplementary material file including: (1) the technical details of the methods, (2) supplementary figures, and (3) a performance measures test.

Abbreviations

BCH-ASD: Boston Children's Hospital Autism Spectrum Disorders cohort; DTH: Data Temporal Heatmap; EHR: Electronic Health Record; ICD: International Classification of Diseases; ICD-9-CM: ICD Ninth Revision, Clinical Modification; IGT plot: Information Geometric Temporal plot; NHDS: National Hospital Discharge Survey; PheWAS: Phenome Wide Association Studies

Acknowledgments

This work was supported by UPV grant PAID-00-17, GVA grant BEST/2018, and projects H2020-SC1-2016-CNECT No. 727560 and H2020-SC1-BHC-2018-2020 No. 825750. The authors thank the community that collaboratively created the Open Source R software and packages used in this work. A special thanks to UpSetR, which inspired our Shiny wrapper landing page.

Competing Interests

The authors declare that they have no competing interests.

References

1. Gewin, V. Data sharing: An open mind on open data. *Nature* 529, 117–119 (2016).
2. Katzan, I. L. & Rudick, R. A. Time to integrate clinical and research informatics. *Sci. Transl. Med.* 4, 162fs41 (2012).
3. Zhu, L. & Zheng, W. J. Informatics, Data Science, and Artificial Intelligence. *JAMA* 320, 1103–1104 (2018).
4. Rajkomar, A., Dean, J. & Kohane, I. Machine Learning in Medicine. *N. Engl. J. Med.* 380, 1347–1358 (2019).
5. Andreu-Perez, J., Poon, C. C. Y., Merrifield, R. D., Wong, S. T. C. & Yang, G.-Z. Big data for health. *IEEE J Biomed Health Inform* 19, 1193–1208 (2015).
6. Sáez, C., Rodrigues, P. P., Gama, J., Robles, M. & García-Gómez, J. M. Probabilistic

change detection and visualization methods for the assessment of temporal stability in biomedical data quality. *Data Min. Knowl. Discov.* 29, 950–975 (2015).

7. Schlegel, D. R. & Ficheur, G. Secondary Use of Patient Data: Review of the Literature Published in 2016. *Yearb. Med. Inform.* 26, 68–71 (2017).
8. Agniel, D., Kohane, I. S. & Weber, G. M. Biases in electronic health record data due to processes within the healthcare system: retrospective observational study. *BMJ* 361, k1479 (2018).
9. Sáez, C. & García-Gómez, J. M. Kinematics of Big Biomedical Data to characterize temporal variability and seasonality of data repositories: Functional Data Analysis of data temporal evolution over non-parametric statistical manifolds. *Int. J. Med. Inform.* 119, 109–124 (2018).
10. Knight, L., Halech, R., Martin, C. & Mortimer, L. *Impact of changes in diabetes coding on Queensland hospital principal diagnosis morbidity data*. (Health Statistics Centre, Queensland Health, 2011).
11. Sáez, C. *et al.* Applying probabilistic temporal and multisite data quality control methods to a public health mortality registry in Spain: a systematic approach to quality control of repositories. *J. Am. Med. Inform. Assoc.* 23, 1085–1095 (2016).
12. Wright, A. *et al.* Best practices for preventing malfunctions in rule-based clinical decision support alerts and reminders: Results of a Delphi study. *Int. J. Med. Inform.* 118, 78–85 (2018).
13. Sugiyama, M., Lawrence, N. D., Schwaighofer, A. & Others. *Dataset shift in machine learning*. (The MIT Press, 2017).
14. Moreno-Torres, J. G., Raeder, T., Alaiz-Rodríguez, R., Chawla, N. V. & Herrera, F. A unifying view on dataset shift in classification. *Pattern Recognit.* 45, 521–530 (2012).
15. Leek, J. T. *et al.* Tackling the widespread and critical impact of batch effects in high-throughput data. *Nat. Rev. Genet.* 11, 733–739 (2010).
16. Goh, W. W. B., Wang, W. & Wong, L. Why Batch Effects Matter in Omics Data, and How to Avoid Them. *Trends Biotechnol.* 35, 498–507 (2017).

17. Centers for Disease Control and Prevention's (CDC), National Center for Health Statistics, U.S. Department of Health & Human Services. International Classification of Diseases,Ninth Revision, Clinical Modification (ICD-9-CM). (2015). Available at: https://www.cdc.gov/nchs/icd/icd9cm.htm. (Accessed: 16th July 2018)
18. Shewhart, W. A. & Deming. W. E. Statistical Method from the Viewpoint of Quality Control. New York, Dover (1986).
19. Westgard, J.O. Basic QC Practices: Training in Statistical Quality Control for Medical Laboratories. Madison, WI (2010).
20. Svolba, G. & Bauer, P. Statistical quality control in clinical trials. *Control. Clin. Trials* 20 (6) 519–530 (1999).
21. Bray, F. & Parkin, D. M. Evaluation of data quality in the cancer registry: principles and methods. Part I: comparability, validity and timeliness. *Eur. J. Cancer*, 45(5), 747-755 (2009).
22. Kahn, M. G., Raebel, M. A., Glanz, J. M., Riedlinger, K., & Steiner, J. F. A pragmatic framework for single-site and multisite data quality assessment in electronic health record-based clinical research. *Med. care.* 50 (2012).
23. Box, G.E., Jenkins, G.M., Reinsel, G.C. & Ljung, G.M. Time Series Analysis: Forecasting and Control, John Wiley & Sons (2015).
24. Springate, D.A., Parisi. R., Olier. I., Reeves, D., Kontopantelis, E. rEHR: An R package for manipulating and analysing Electronic Health Record data. PLoS ONE 12(2): e0171784 (2017).
25. Choi, L., Carroll, R. J., Beck, C., Mosley, J. D., Roden, D. M., Denny, J. C., & Van Driest, S. L. Evaluating statistical approaches to leverage large clinical datasets for uncovering therapeutic and adverse medication effects. Bioinformatics, 34(17), 2988-2996 (2018).
26. Gutiérrez-Sacristán, A., Bravo, À., Giannoula, A., Mayer, M. A., Sanz, F., & Furlong, L. I. comoRbidity: an R package for the systematic analysis of disease comorbidities. Bioinformatics, 34(18), 3228-3230. (2018).

27. Tsay, R. S. Multivariate time series analysis: with R and financial applications. John Wiley & Sons. (2013).
28. Scrucca, L. qcc: an R package for quality control charting and statistical process control. R News 4/1, 11-17. (2004).
29. Denny, J. C. *et al.* Systematic comparison of phenome-wide association study of electronic medical record data and genome-wide association study data. *Nat. Biotechnol.* 31, 1102–1110 (2013).
30. Khera, R., Dorsey, K. B. & Krumholz, H. M. Transition to the ICD-10 in the United States: An Emerging Data Chasm. *JAMA* 320, 133–134 (2018).

Figure 1 (png) Click here to access/download;Figure;Figure_1.png

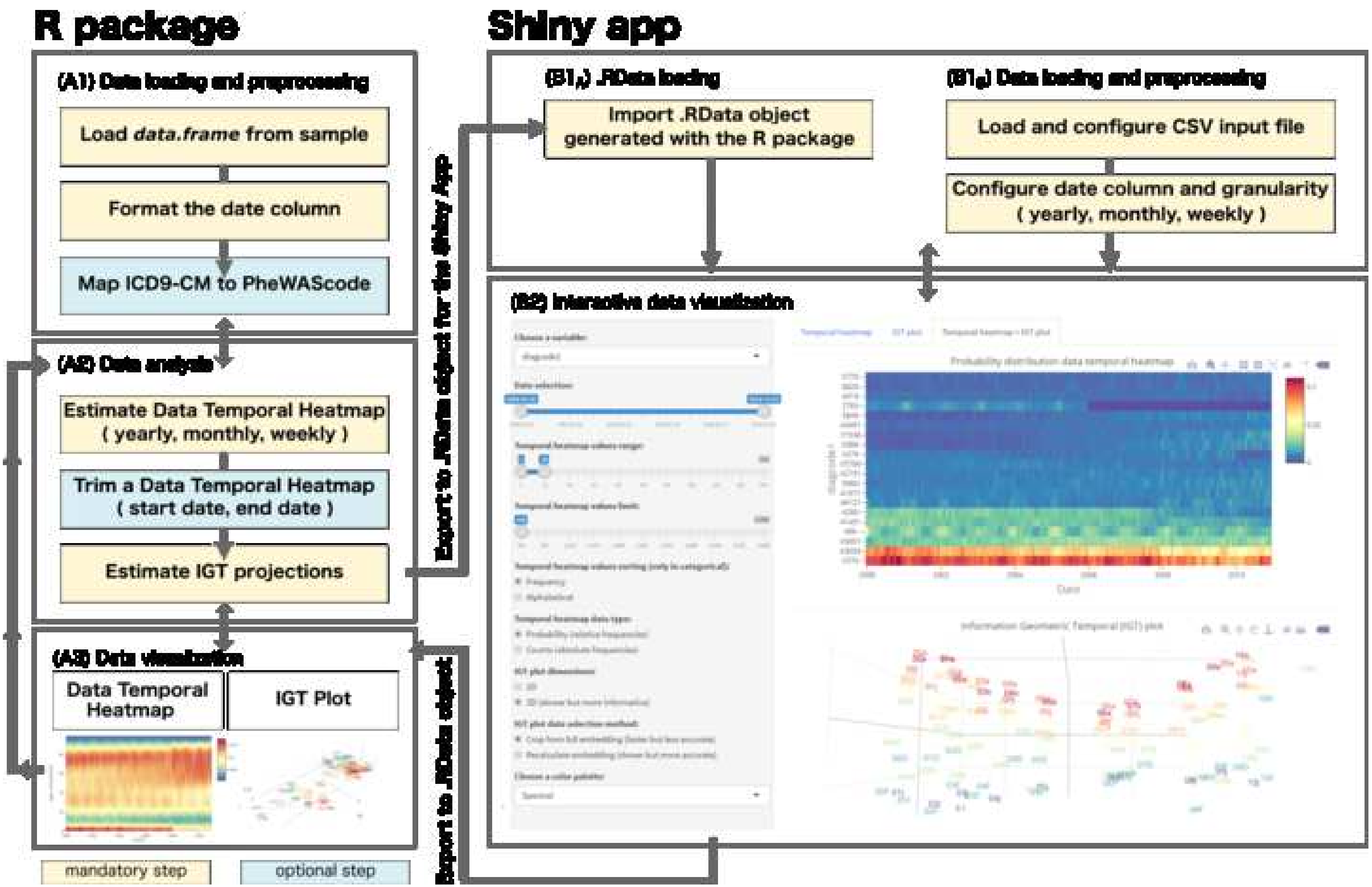

Figure 2 (png) Click here to access/download;Figure;Figure_2.png

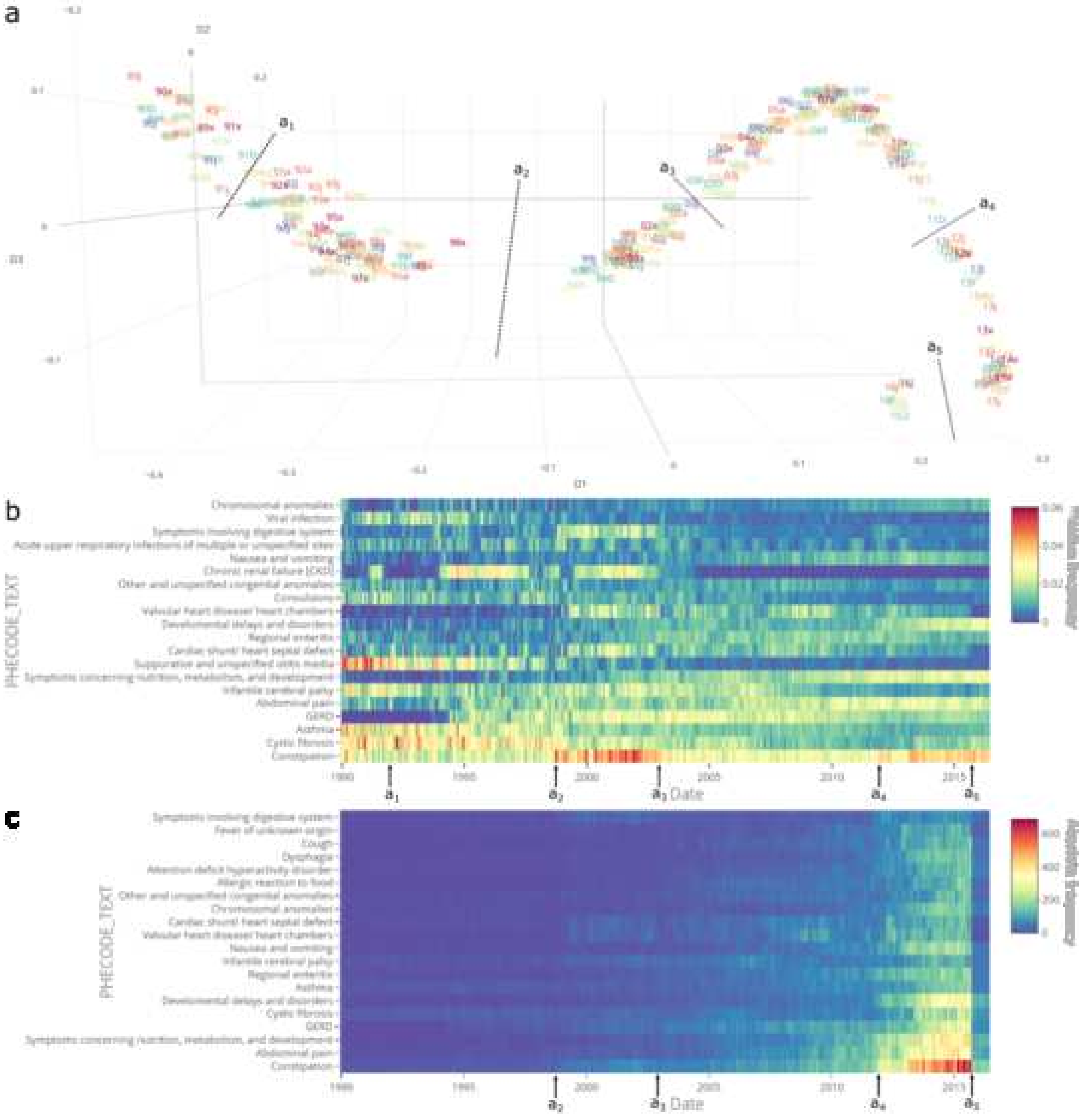

Figure 3 (png) Click here to access/download;Figure;Figure_3.png

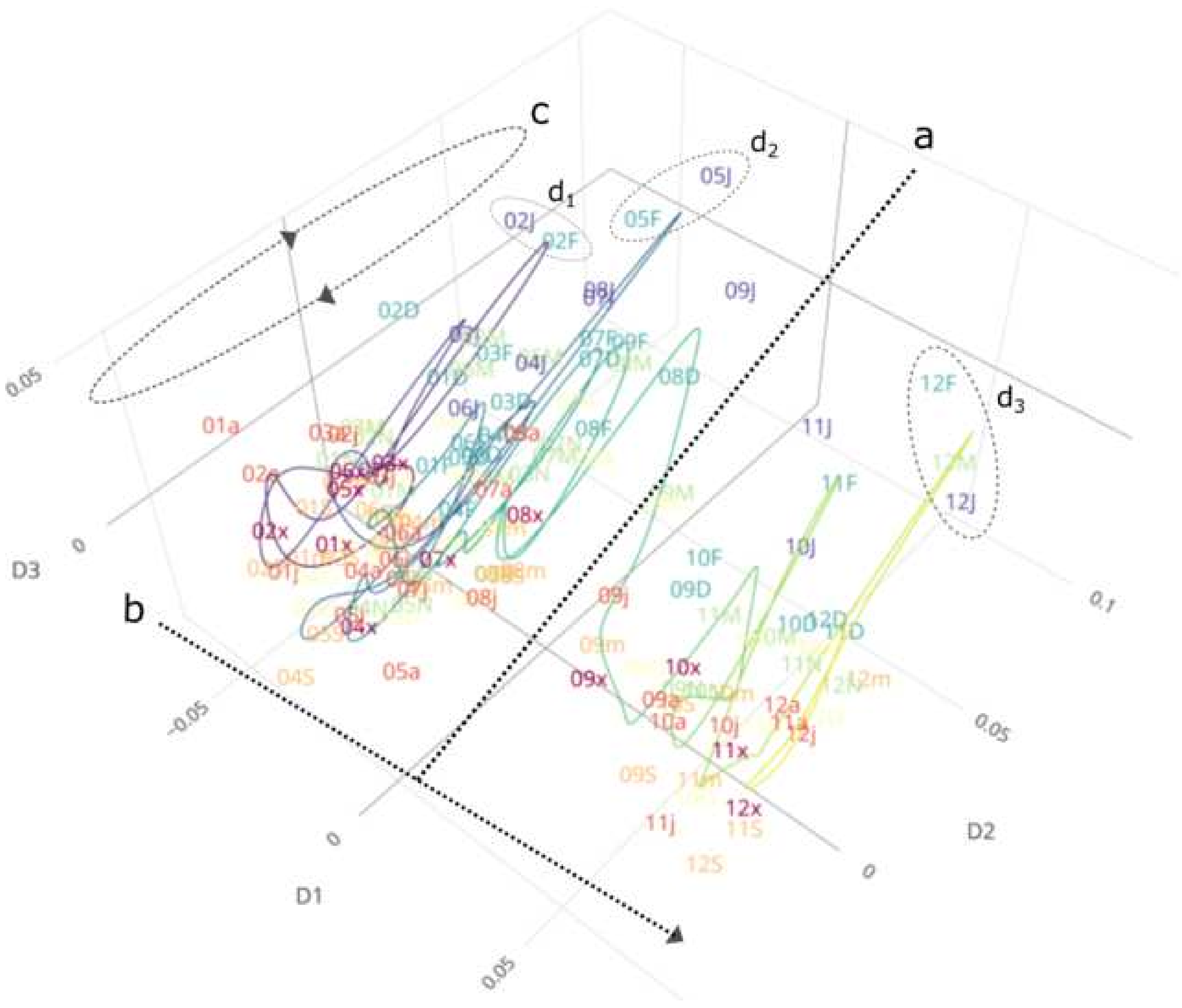

Figure 1 (vectorial-svg)

Click here to access/download
Supplementary Material
Figure_1.svg

Figure 2 (vectorial-svg)

Click here to access/download
Supplementary Material
Figure_2.svg

Figure 3 (vectorial-svg)

Click here to access/download
Supplementary Material
Figure_3.svg

Click here to access/download
Supplementary Material
2020_ehrtemporalvariability_saez_et_al_supp_material_v2.docx

Experiment on p-values on the NHDS dataset, complementing
answer to question R2Q3 by Reviewer 2

Click here to access/download
Supplementary Material
variabilityPValuesTestOnNHDS.R
