# Supplementary material for: EHRtemporalVariability: delineating temporal data-set shifts in electronic health records
Source: Gigascience. 2020 Jul 30;9(8):giaa079. doi: 10.1093/gigascience/giaa079 (PMC7391413; doi:10.1093/gigascience/giaa079)
Supplement: giaa079_Supplemental_Files [file giaa079_supplemental_files.zip › 2020_ehrtemporalvariability_saez_et_al_supp_material_v2.docx]

EHRtemporalVariability: delineating temporal dataset shifts in electronic health records

**SUPPLEMENTARY MATERIAL**

**Carlos Sáez,^1,2,^* Alba Gutiérrez-Sacristán,^2^ Isaac Kohane,^2^ Juan M García-Gómez,^1,†^ Paul Avillach^2,†^**

^1^ Biomedical Data Science Lab, Instituto Universitario de Tecnologías de la Información y Comunicaciones (ITACA), Universitat Politècnica de València (UPV), Camino de Vera s/n, Valencia 46022, España

^2^ Department of Biomedical Informatics, Harvard Medical School, Boston, MA, USA

* Corresponding author <carsaesi@upv.es>

^†^ Both to be regarded as last authors

**Contents**

[1. Technical development of the method 2](#_Toc22729102)

[2. Supplementary figures 4](#_Toc22729103)

[3. Performance measures 5](#_Toc22729104)

# Technical development of the method

**Input**

The input of the variability assessment method, and so of the EHRtemporalVariability package, is an N-by-V matrix *X,* where N is the number of individuals and V the number of variables. An example input is built into the package CRAN and GitHub repositories and described in provided documentation. We define *x_iv_* as the variable *v* for individual *i*. One of the variables, let y where [
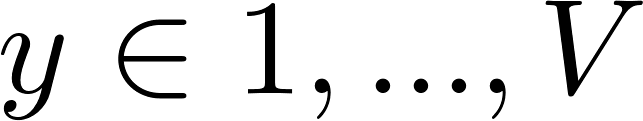
](https://www.codecogs.com/eqnedit.php?latex=y%5Cin%201%2C%20...%2C%20V%250), must represent the reference date of each individual *x_i_* who would be used in the batching process, consequently V≥2. The remainder,[
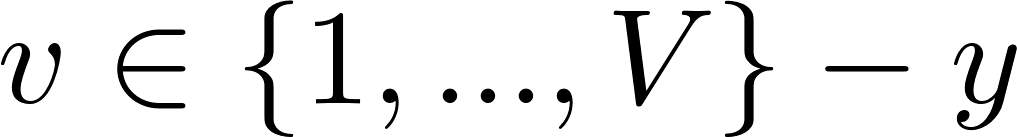
](https://www.codecogs.com/eqnedit.php?latex=v%5Cin%20%5C%7B1%2C%20...%2C%20V%5C%7D-y%250), represents variables for which temporal variability is analyzed. Types for these variables include categorical (e.g., phenotypes, declared as “*factor”* or “*character”* in R); numerical discrete (e.g., patient age in years, declared as “*integer”*); and numerical continuous (e.g., lab results, declared as “*numerical”*).

**Batching**

The batching granularity is selected at the level of yearly, monthly, or weekly. Specifically, *X* is partitioned into temporary consecutive batches
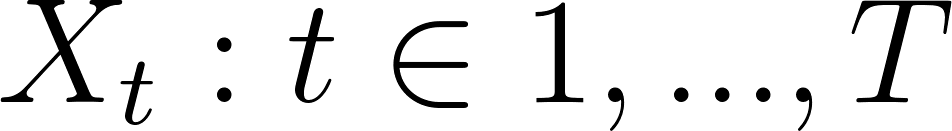
, where T is the total number of time batches, beginning at the first and extending to the last date in variable *x_i_*_y_. Note that the sample size *N*_t_ of each batch can be different throughout the set of *X*_t_, depending on the availability of individuals in the original data. In the case in which there are no individuals within a specific date range, the corresponding batch *X*_t_ is set as the empty set ∅.

**Estimation of Data Temporal Maps**

A DTH is defined as a T-by-B matrix *M*, where *M_tb_* is the relative frequency of individuals at the time batch *t* that fall within the distribution support, defined by the bin *b*. For a given variable *v*, the row *M_t_* corresponds to the probability distribution of the data in batch *X_t_*, which estimation and binning scheme is defined according to the selected type of the variable, as follows:

**Categorical variables**

For categorical variables, the distributions *M_t_* are defined by the frequency points *f_1_*,...,*f*_B_, associated with each of the categories [
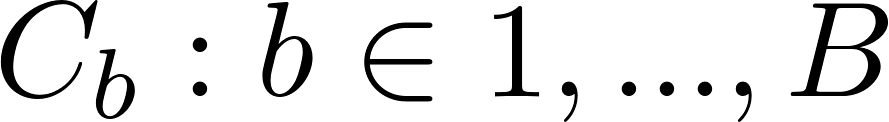
](https://www.codecogs.com/eqnedit.php?latex=C_b%3Ab%5Cin%7B1%2C...%2CB%7D%250) in *X_v_* (e.g., the 1645 different PheWAS codes), where [
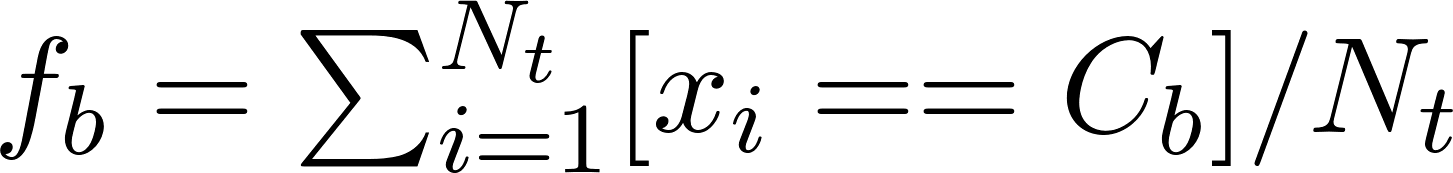
](https://www.codecogs.com/eqnedit.php?latex=f_b%20%3D%20%5Csum%5E%7BN_t%7D_%7B%7Bi%7D%3D1%7D%20%5Bx_i%3D%3DC_b%5D%2FN_t%250) and [·] is the Iverson bracket.

**Numerical discrete variables**

For numerical discrete variables, the distributions *M_t_* are defined by the frequency points *f_b_*,...,*f*_B_, associated with each of the *I*_b_ consecutive B natural numbers between [*min*(*x_v_*), *max*(*x_v_*)], where [
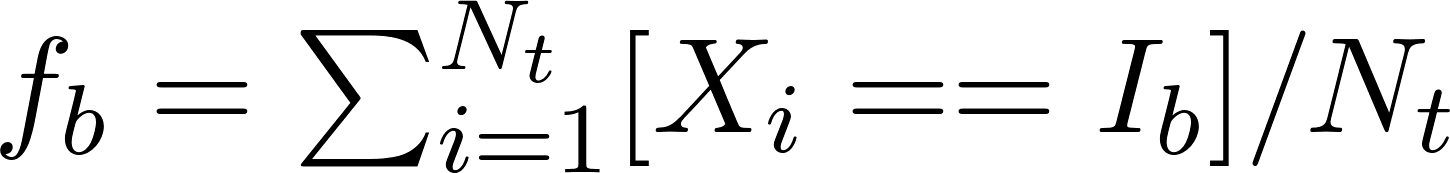
](https://www.codecogs.com/eqnedit.php?latex=f_b%20%3D%20%5Csum%5E%7BN_t%7D_%7Bi%3D1%7D%20%5BX_i%3D%3DI_b%5D%2FN_t%250).

**Numerical continuous variables**

For numerical continuous variables, the distributions *M_t_* are defined by the frequency points *f*_1_,...,*f*_B,_ associated with the breaking points *q_1_*,...,*q*_B+1_, dividing the distribution support into B equidistant bins between [*min*(*x_v_*), *max*(*x_v_*)]. In this instance, we can set a specific value for B or use the default value of 100 bins. Next, we can choose to obtain the distribution from a binned histogram, in which [
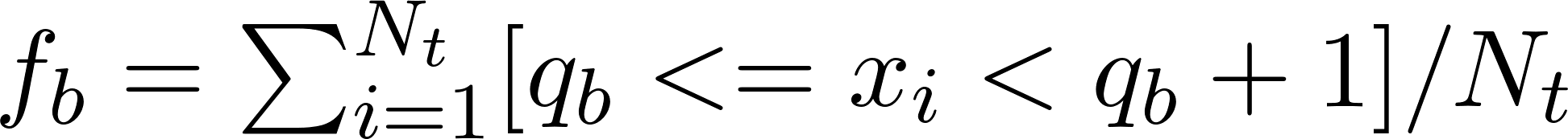
](https://www.codecogs.com/eqnedit.php?latex=f_b%20%3D%20%5Csum%5E%7BN_t%7D_%7Bi%3D1%7D%20%5Bq_b%3C%3Dx_i%7D%3Cq_b%2B1%5D%2FN_t%250), or use a smoothed Kernel Density Estimation (KDE) [20] of the distribution. In the KDE case
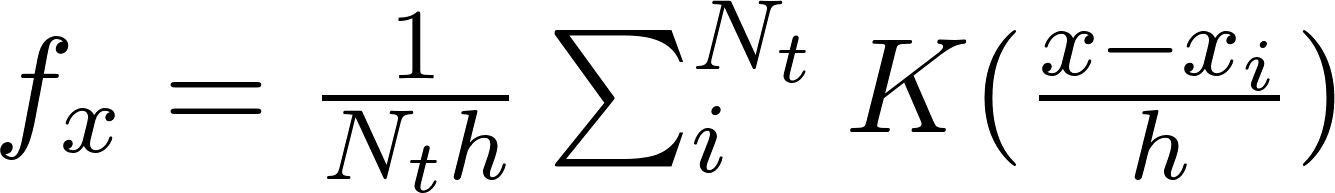
, where *x* is the center of a bin, *K*(·) is a Gaussian kernel function, *h* is the bandwidth (using the Silverman’s 1986 default [21]).

A naturally integer variable can optionally be declared as “*numerical”* in R in order to estimate its distribution according to the numerical continuous scheme. This can be either as a binned or smoothed histogram, e.g., an integer variable with a possible large range, such as a length of stay in days of hospital admissions.

For visualizing both relative and absolute frequencies in DTHs, an absolute version of *M,* is also stored in addition to the relative that is used in IGT plot estimation. In the cases in which *X_t_* = ∅, no distribution can be estimated. In such a case, we provide two ways of filling these temporal gaps in *M*: using *NA* values or a linear interpolation over *t* on *M_tb_*. This choice leads to different results in the resultant visualizations. Therefore, we recommend using first the *NA* (the default) to better highlight those gaps and then try the smoothing option.

**Estimation of Information Geometric Temporal plots**

IGT plots project data time batches as a series of points, whereby the distances among them correspond to the dissimilarity of their statistical distributions, namely, a non-parametric temporal statistical manifold. The IGT plot of a variable *v* is estimated by means of embedding it into a Euclidean space. The batched statistical distributions of *v* are depicted as recorded in the corresponding DTH *M*. First, a T-by-T symmetric dissimilarity matrix *Y* is calculated, compiling the
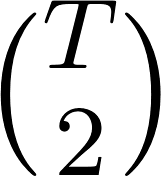
 pairwise distances between the distributions among the time batches. EHRtemporalVariability currently uses the Jensen-Shannon-Distance [[22, 23]](https://upvedues-my.sharepoint.com/personal/carsaesi_upv_edu_es/Documents/Papers/2018%20EHRtemporalVariability/2018_ehrtemporalvariability_natmethodsbriefcomm.docx#_msocom_1) as a dissimilarity metric between distributions, where a distance of 0 means equal distributions, and 1 means non-overlapping probability masses:

[
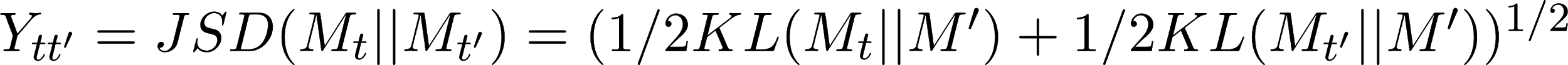
](https://www.codecogs.com/eqnedit.php?latex=Y_%7Btt'%7D%20%3D%20JSD(M_t%7C%7CM_%7Bt'%7D)%20%3D%20(%7B1%2F2%7D%20KL(M_t%7C%7CM')%20%2B%20%7B1%2F2%7D%20KL(M_%7Bt'%7D%7C%7CM'))%5E%7B1%2F2%7D%250), where *Y_tt’_* is the JSD between time batches *t* and *t’*; *KL*(*P*|*Q*) is the Kullback-Leibler divergence [24] between arbitrary distributions: P and Q as [
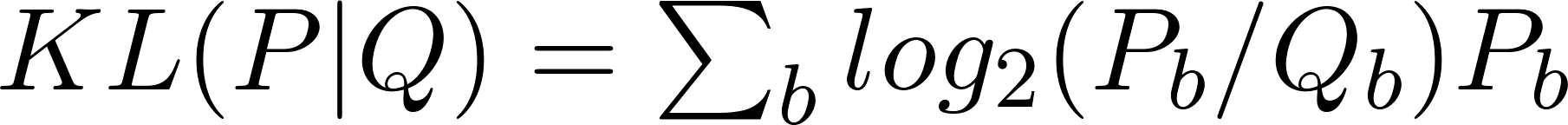
](https://www.codecogs.com/eqnedit.php?latex=KL(P%7CQ)%20%3D%20%5Csum_b%20log_2(P_b%2FQ_b)P_b%250); and *P_b_* and *Q_b_* are the probability masses at bin b, and [
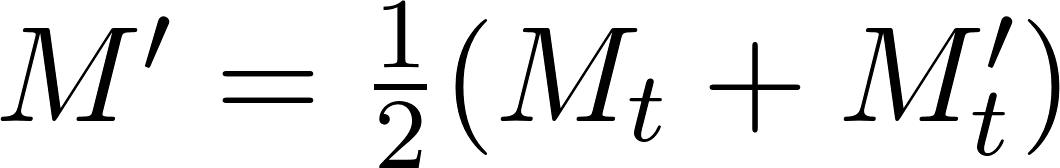
](https://www.codecogs.com/eqnedit.php?latex=M'%20%3D%20%5Cfrac%7B1%7D%7B2%7D%20(M_t%20%2B%20M_t')%250).

Next, the dissimilarity matrix *Y* is embedded using Multidimensional Scaling (MDS) [25] into the set of points *P*, where *P* is a T-by-D matrix, and *P_td_* corresponds the coordinate of time batch *t* in the *d_th_* dimension. The user can choose between classical and non-metric MDS. D can be chosen by the user, requiring [
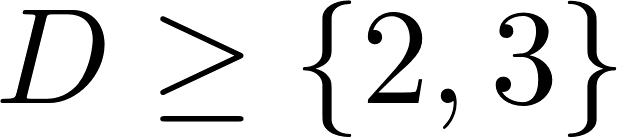
](https://www.codecogs.com/eqnedit.php?latex=D%5Cgeq%5C%7B2%2C3%5C%7D%250) for further visualization. The embedded dimensions can be understood as sorted based on explained variance. Therefore, plotting the first two or three dimensions shows the largest variability components over time batches. Further, dimensions can be obtained for further analysis. In IGT plots, batches are labeled with their date and colored in order to distinguish seasonal effects.

***Technical references***

[20] Parzen E. On estimation of a probability density function and mode, Ann. Math. Stat. **33** (3) 1065–1076 (1962).

[21] Silverman, Bernard W. Density estimation for statistics and data analysis. Chapman & Hall/CRC. (1986)

[22] Lin, J. Divergence measures based on the Shannon entropy. IEEE Trans. Inf. Theory **37** 145–151 (1991).
[23] Endres D, Schindelin J. A new metric for probability distributions, IEEE Trans. Inf.
Theory **49** (7) 1858–1860 (2003).

[24] Kullback S, Leibler R.A. On information and sufficiency. Ann. Math. Stat. **22** (1) 79–86 (1951).
[25] Torgerson W. Multidimensional scaling: I. Theory and method. Psychometrika **17** (4) 401–419 (1952).

# Supplementary figures


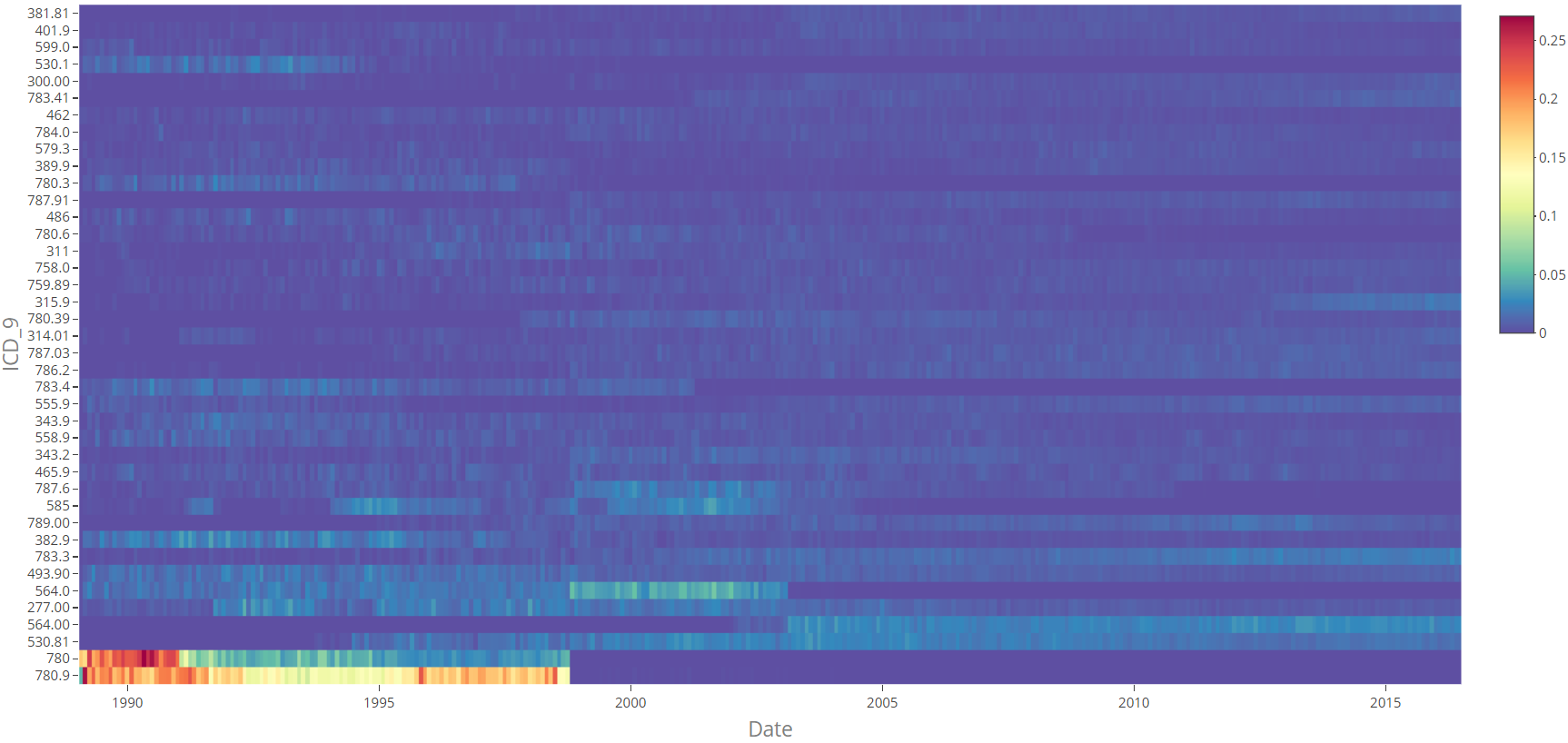


**Supplementary material Fig 1**. Data Temporal Heatmap of relative frequencies of ICD-9-CM codes of the BCH-ASD case study (40 most prevalent).


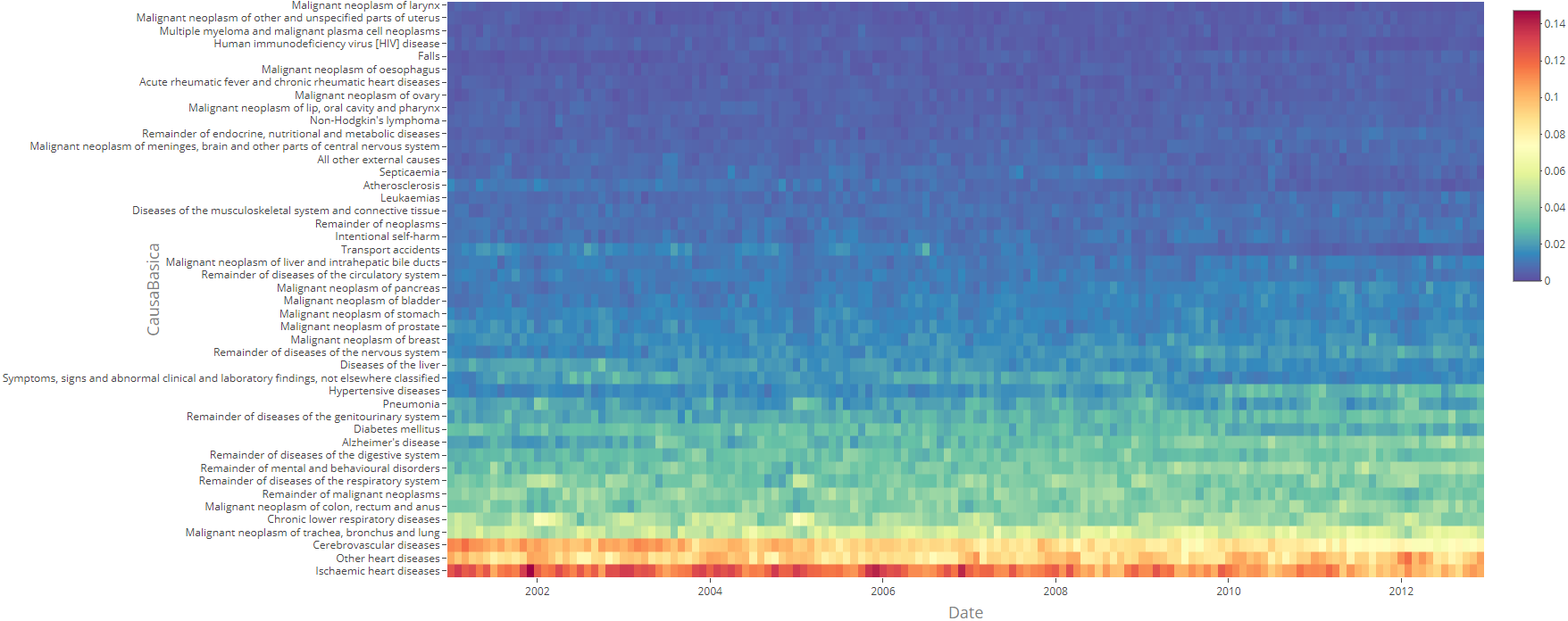


**Supplementary material Fig 2**. Data Temporal Heatmap of relative frequencies of the Basic Cause of Death in the Mortality Registry of the Region of Valencia, Spain (45 most prevalent).

# Performance measures

We provide next a comparative reference for performance measures of the EHRtemporalVariability in estimating Data Temporal Heatmaps (DTHs) and Information Geometric Temporal (IGT) projections. We took measurements in a single computing thread of a regular laptop. The benchmark computer was an Intel Core i7-6700HQ CPU @ 2.60GHz with 16GB of RAM. Tests consisted of benchmarking the *estimateDataTemporalMap* and *estimateIGTProjection* functions on the three case studies, BCH-ASD, MORTALITY, and NHDS, each with different temporal granularity levels: yearly, monthly, and weekly. (Note: the maximum detail of NHDS dates was monthly, prohibiting weekly batching). Tests were repeated 10 times, and the average time elapsed was obtained as a performance measure. Results are provided in Tables SM1 and SM2.

The interpretation of the benchmark results was supported by the features of each dataset. Table SM3 describes general dataset features, and tables SM4 to SM6 describe the variables contained in each dataset. The time elapsed for DTH estimation (Table SM1) appeared to be dependent on the function of the number of individuals of the dataset and the time granularity for the analysis. The latter was related to the number of batches, and the former to the individuals being used for the distribution estimation at each batch. The estimation of time elapsed for IGT projections appeared to be dependent on the function of the number of temporal batches—and therefore, temporal granularity of the analysis—and particularly on the number of categories/bins present in the variables. These factors implied a higher computational cost in the calculus of the dissimilarity matrix of Jensen-Shannon distances and in the posterior Multi-Dimensional Scaling embedding.

**Table SM1**. Performance measures of the estimation of Data Temporal Heatmaps (DTHs), using the *estimateDataTemporalMap* function of EHRtemporalVariability.

| **Case study** | **Temporal granularity** | **Average time elapsed**  **(10 repetitions)** |
| --- | --- | --- |
| BCH-ASD | Yearly | 1.14 sec |
| BCH-ASD | Monthly | 1.78 sec |
| MORTALITY | Yearly | 1.84 sec |
| MORTALITY | Monthly | 3.48 sec |
| BCH-ASD | Weekly | 5.23 sec |
| MORTALITY | Weekly | 10.20 sec |
| NHDS | Yearly | 17.18 sec |
| NHDS | Monthly | 18.56 sec |

**Table SM2**. Performance measures of the estimation of Information Geometric Temporal (IGT) projections, using the *estimateIGTProjection* function of EHRtemporalVariability.

| **Case study** | **Temporal granularity** | **Average time elapsed**  **(10 repetitions)** |
| --- | --- | --- |
| MORTALITY | Yearly | 0.12 sec |
| BCH-ASD | Yearly | 0.37 sec |
| NHDS | Yearly | 0.41 sec |
| MORTALITY | Monthly | 13.84 sec |
| BCH-ASD | Monthly | 43.90 sec |
| NHDS | Monthly | 47.65 sec |
| MORTALITY | Weekly | 4 min 8.07 sec |
| BCH-ASD | Weekly | 13 min 40.40 sec |

**Table SM3**. General features of the three case studies: Boston Children’s Hospital Autism Spectrum Disorders cohort (BCH-ASD), Mortality Registry of the Region of Valencia, Spain (MORTALITY), National Hospital Discharge Survey (NHDS) dataset.

| **Case study** | **N** | **Num. vars.** | **Cat. vars.** | **Date from** | **Date to** | **Years** | **Months** | **Weeks** |
| --- | --- | --- | --- | --- | --- | --- | --- | --- |
| BCH-ASD | 1,194,113 | 0 | 2 | Jul 1981 | Jul 2016 | 36 | 421 | 1823 |
| MORTALITY | 469,356 | 2 | 21 | Jan 2001 | Dec 2012 | 12 | 144 | 627 |
| NHDS | 3,257,718 | 4 | 30 | Jan 2000 | Dec 2010 | 11 | 132 | NA |

**Table SM4**. Description of variables of the Boston Children’s Hospital Autism Spectrum Disorders (BCH-ASD) cohort.

| **Variable name** | **Description** | **Type** | **Unique codes / bins** |
| --- | --- | --- | --- |
| *ICD-9* | ICD-9-CM code | Categorical | 7350 |
| *PHECODE_TEXT* | PheWAS code | Categorical | 1754 |

**Table SM5**. Description of variables of the Mortality Registry of the Region of Valencia, Spain (MORTALITY).

| **Variable name** | **Description** | **Type** | **Unique codes / bins** |
| --- | --- | --- | --- |
| *FcNac* | Date of birth | Date (numerical) | 100 |
| *Sexo* | Gender | Categorical | 2 |
| *MuniResAnon* | City of residence | Categorical | 541 |
| *MuniDefAnon* | City of death | Categorical | 538 |
| *LugarDef* | Location at death | Categorical | 6 |
| *HoraDef* | Time of death | Categorical | 1463 |
| *CausaBasica* | Basic cause of death (ICD-10 List 1) | Categorical | 83 |
| *CausaInmediat1* | Immediate cause of death 1 (ICD-10 List 1) | Categorical | 73 |
| *CausaInmediat2* | Immediate cause of death 2 (ICD-10 List 1) | Categorical | 71 |
| *CausaInmediat3* | Immediate cause of death 3 (ICD-10 List 1) | Categorical | 58 |
| *CausaIntermed1* | Intermediate cause of death 1 (ICD-10 List 1) | Categorical | 79 |
| *CausaIntermed2* | Intermediate cause of death 2 (ICD-10 List 1) | Categorical | 75 |
| *CausaIntermed3* | Intermediate cause of death 3 (ICD-10 List 1) | Categorical | 63 |
| *CausaInicial1* | Initial cause of death 1 (ICD-10 List 1) | Categorical | 82 |
| *CausaInicial2* | Initial cause of death 2 (ICD-10 List 1) | Categorical | 76 |
| *CausaInicial3* | Initial cause of death 3 (ICD-10 List 1) | Categorical | 70 |
| *CausaContribu1* | Contributive cause of death 1 (ICD-10 List 1) | Categorical | 78 |
| *CausaContribu2* | Contributive cause of death 2 (ICD-10 List 1) | Categorical | 72 |
| *CausaContribu3* | Contributive cause of death 3 (ICD-10 List 1) | Categorical | 71 |
| *MedicoAnom* | Doctor | Categorical | 18657 |
| *AgeDefY* | Age at death | Numerical (integer) | 100 |
| *ProvDep* | Province | Categorical | 3 |
| *Departamento* | Health department | Categorical | 24 |

**Table SM6**. Description of variables of the National Hospital Discharge Survey (NHDS) dataset.

| **Variable name** | **Description** | **Type** | **Unique codes / bins** |
| --- | --- | --- | --- |
| *age-smoothed* | Age at discharge | Numerical (smoothed using KDE) | 100 |
| *age-integer* | Age at dischrage | Numerical (integer) | 100 |
| *sex* | Gender | Categorical | 2 |
| *newborn* | Newborn flag | Categorical | 2 |
| *race* | Race | Categorical | 8 |
| *marital* | Marital status | Categorical | 6 |
| *disstatus* | Discharge status | Categorical | 7 |
| *dayscare* | Days of stay | Numerical | 100 |
| *lengthflag* | Flag of length of stay | Categorical | 2 |
| *region* | Region code | Categorical | 4 |
| *hospbeds* | Hospital beds | Integer | 5 |
| *hospownership* | Hospital ownership | Categorical | 3 |
| *diagcode1* | Diagnosis at discharge 1 (ICD-9-CM) | Categorical | 8625 |
| *diagcode2* | Diagnosis at discharge 2 (ICD-9-CM) | Categorical | 9602 |
| *diagcode3* | Diagnosis at discharge 3 (ICD-9-CM) | Categorical | 9511 |
| *diagcode4* | Diagnosis at discharge 4 (ICD-9-CM) | Categorical | 9201 |
| *diagcode5* | Diagnosis at discharge 5 (ICD-9-CM) | Categorical | 8809 |
| *diagcode6* | Diagnosis at discharge 6 (ICD-9-CM) | Categorical | 8319 |
| *diagcode7* | Diagnosis at discharge 7 (ICD-9-CM) | Categorical | 7932 |
| *proccode1* | Procedure code 1 (ICD-9-CM) | Categorical | 3260 |
| *proccode2* | Procedure code 2 (ICD-9-CM) | Categorical | 3219 |
| *proccode3* | Procedure code 3 (ICD-9-CM) | Categorical | 3013 |
| *proccode4* | Procedure code 4 (ICD-9-CM) | Categorical | 2729 |
| *princpayment* | Principal source of payment | Categorical | 11 |
| *secondpayment* | Secondary source of payment | Categorical | 10 |
| *drg* | Diagnosis Related Group | Categorical | 863 |
| *diagcode1-phewascode* | Diagnosis at discharge 1 (PheWAS code) | Categorical | 1696 |
| *diagcode2-phewascode* | Diagnosis at discharge 2 (PheWAS code) | Categorical | 1762 |
| *diagcode3-phewascode* | Diagnosis at discharge 3 (PheWAS code) | Categorical | 1776 |
| *diagcode4-phewascode* | Diagnosis at discharge 4 (PheWAS code) | Categorical | 1762 |
| *diagcode5-phewascode* | Diagnosis at discharge 5 (PheWAS code) | Categorical | 1769 |
| *diagcode6-phewascode* | Diagnosis at discharge 6 (PheWAS code) | Categorical | 1765 |
| *diagcode7-phewascode* | Diagnosis at discharge 7 (PheWAS code) | Categorical | 1748 |
