# Supplementary material for: EHRtemporalVariability: delineating temporal data-set shifts in electronic health records
Source: Gigascience. 2020 Jul 30;9(8):giaa079. doi: 10.1093/gigascience/giaa079 (PMC7391413; doi:10.1093/gigascience/giaa079)
Supplement: giaa079_Response_to_Reviewer_Comments_Original_Submission [file giaa079_response_to_reviewer_comments_original_submission.pdf]

Author's Response To Reviewer Comments

Close

Dear Editor,

Please find enclosed our revised version of the manuscript entitled, “EHRtemporalVariability: delineating temporal dataset shifts in electronic health records" (GIGA-D-19-00376).

We would like to thank the Editor for the opportunity to resubmit the manuscript to be considered for publication in GIGASCIENCE and also the Reviewers for their comments. We have used them to improve both the manuscript and the application.

Please find below our response to the Reviewers’ comments.

Editor

EQ1. In addition, please register any new software application in the bio.tools and SciCrunch.org databases to receive RRID (Research Resource Identification Initiative ID) and biotoolsID identifiers, and include these in your manuscript. This will facilitate tracking, reproducibility and re-use of your tool.

According to the Editor’s request, we have registered our software in bio.tools (https://bio.tools/ehrtemporalvariability) and it is approved as a new resource in SciCrunch.org (RRID:SCR_018663). We thank the Editor for making this suggestion; we are sure this will facilitate disseminating this work to end users.

Reviewer reports

Reviewer #1

"The main objective of this technical note is to introduce an R package and a Shiny app that was developed to explore and identify the temporal variability of electronic health record (EHR). To achieve this goal, the authors first gave a brief introduction to the methodologies embedded in the R tools in a way that easy to understand and elucidated the functionalities of these tools. Three case studies were listed afterward. This topic is in-time for promoting the usage of EHR in clinical research. However, it could be further improved if the following comments are soundly fixed.

-We thank the Reviewer for highlighting the timeliness of our work for clinical research. We also expect that the work will help facilitate the performance of reliable Real World Data research using large datasets acquired over long time periods."

R1Q1) "The paper only shows the IGT plot and temporal heatmap, it would be improved if the authors provide more comprehensive analysis functions and visualizations shown in the original case studies, e.g. the kinematic figures, temporal subgroup discovery and clustering validation, the variability of JSD distributions."

In this work—and upon the current release of the tool—we aimed to provide full support on IGT plots and temporal heatmap estimation and visualization. We also planned for further versions of the tool, including improved functionality for the kinematic figures described in our IJMI paper and additional subgroup discovery support. However, following the Reviewer's suggestion, we have further added the ability to estimate and plot the kinematic trajectory to the IGT projection. The former is done using the

new function “estimateIGTTrajectory” and the latter using the new parameter “trajectory” in the plotIGTProjection function. We refer to the Reviewer to the 2-D sample in the new package Vignette: http://personales.upv.es/carsaesi/EHRtemporalVariability/EHRtemporalVariability.html#trajectory, as well as to the online demo at http://ehrtemporalvariability.upv.es.

In addition, we added an extra example code to the Vignette to validate the temporal clusters using the DBSCAN clustering algorithm. Specifically, we added a new section in the Vignette (Section 5) to help interpreting temporal changes in IGT projections:
http://personales.upv.es/carsaesi/EHRtemporalVariability/EHRtemporalVariability.html#example

R1Q2) "The manuscript needs careful editing for English and style. For example, the arrows and result image A1-A3 are not vertically aligned well in Figure 1; the arrow in A2 uses a lighter grey color while the meaning seems no different from others; the different border width of B2; the local version of Shiny app accepts input of three separators as comma, semicolon, and tab, however, the authors only claim the input as comma-separated values file (CSV)."

Responding to the Reviewer’s comment, we have carefully revised Figure 1. We are aware of the CSV claim, however, we chose to use it instead of the alternate delimiter-separated values (DSV), given that CSV is more widely accepted and equivalent in the sense that it also accepts using distinct delimiter values (i.e., semicolons and commas are distinctly used across countries). Finally, the manuscript was revised by a professional proofreading service in American English. To address the Reviewer’s comment specifically, however, we edited an additional time for this revised version.

R1Q3) The abbreviation of National Hospital Discharge Survey in the title of table SM3&6 is incorrect.

We thank the Reviewer for pointing this out. We have fixed the error accordingly.

Reviewer #2:

"The manuscript presents an EHRtemporalVariability, an R package and accompanying Shiny app, that provides several visualization functions of EHR data towards identifying changes in the distribution of features in the data. The package accepts the data as an R data frame and allows generating Information-Geometric-Temporal (IGT) plots and Data Temporal Heatmaps (DTHs) -two types of plots previously developed by the authors— that help visually detecting different type of changes in the data described as trend, abrupt change, temporal subgroup, seasonality in the manuscript. Overall, the article is well written and the package and the app are fairly explained. I list several points that need to be addressed in my opinion."

We thank the Reviewer for comments about our paper and the app.

R2Q1) "The introduction needs substantial language & organization revision. The authors use "this/these" in many sentences, making hard to follow what exactly being referred in each sentence. Some examples are "These efforts …", "This is similar …", "These artifacts …". Furthermore, the introduction lacks a brief description of the existing methods mentioned by the authors (refs 8-14) as well as other relevant R packages such as EHR, rEHR, cleanEHR."

Regarding the Reviewer's first comment, we would like to note that the manuscript was revised by a professional proofreading service in American English. To address the Reviewer’s particular issues, however, we performed another round of editing on this revised version. Special attention has been given to the pronouns (where we originally aimed to reduce the number of words). We have substituted the specific target nouns.

Regarding the Reviewer’s suggestion of a brief description of other relevant R packages, we have inserted a brief section, where EHR and rEHR are mentioned. Since the cleanEHR package is no longer available in CRAN, we did not include it in our existing method description. We also added to the description some packages focused on time-series analysis, that although not focused on EHR data, could help in the detection of dataset shifts in EHR. The new paragraph (last paragraph in Background) reads as follows:

'In the R programming language, there are distinct packages that can help in managing or describing

EHRs. For example, the rEHR package focuses on querying and filtering, while the EHR and comoRbidity packages allow the performance of descriptive, PheWAS, and comorbidity analyses.[24-26] Other packages, such as MTS or qcc, allow the performance of time-series or SPC analyses, which assist in detecting dataset shifts in EHRs.[27,28]

To our knowledge EHRtemporalVariability is the first package which provides specific dataset shift delineation, which can be used on raw EHRs and other data sources. The key advantage is its suitability for multi-modal and highly coded information, common features of biomedical data.'

Regarding the brief description of our previous methods (references 6, 9 and 11), we included those in the Methods section (second and third paragraph). Given the “Technical Note” style of this manuscript, we decided to place that information in Methods, as it would guide the consequent description of software functionalities.

R2Q2) "In the introduction the manuscript refers to existing methods based on statistical process control and time-series analysis but does not provide the references for these studies. It would be crucial to also add a short text on the contribution of this study (the proposed package) and how it relates / differs from the existing work to position the contribution of the study."

We thank the Reviewer for this suggestion. In response, we have first included the additional references to existing methods based on SPC and time-series (new references 18-23). We have also added the proposed short texts in the package CRAN and GitHub descriptions to better position the contribution of the study.

New CRAN description:
'Functions to delineate temporal dataset shifts in Electronic Health Records through the projection and visualization of dissimilarities among data temporal batches. This is done through the estimation of data statistical distributions over time and their projection in non-parametric statistical manifolds, uncovering the patterns of the data latent temporal variability. EHRtemporalVariability is particularly suited to multi-modal data and categorical variables with a high number of values, common features of biomedical data where traditional statistical process control or time-series methods may not be suitable. Dataset shifts can be explored and identified through visual analytics formats such as Data Temporal heatmaps and Information Geometric Temporal (IGT) plots. An additional 'EHRtemporalVariability' Shiny app can be used to load and explore the package results and even to allow the use of these functions to those users non-experienced in R coding. Preprint published in medRxiv (Sáez et al. 2020) .'

New GitHub description:
Please see https://github.com/hms-dbmi/EHRtemporalVariability#background

R2Q3) "In most case studies, the authors suggest that there was no apparent cause for the shift. It would certainly be useful to assign a confidence/statistical score (such as p-value/confidence interval) regarding the extremeness of observing the shit in concern. Moreover, as far as I understand the projection involves data compression due to the choice of the 2-3 dimensions to be displayed, it would also be useful to know the percentage of the information the visually presented plot captures (e.g., analogous to variant explained in a PCA plot)."

Although in the Mortality case study, the causes of the shifts are clearly determined (we refer the Reviewer to our previous paper in JAMIA), we found it more challenging to determine the exact cause in the BCH-ASD and NHDS cases. We did, however, discuss some possibilities of causes that are related to changes/updates in coding practices.

We concede the Reviewer’s valid point in terms of the confidence/statistical score aspect. We have discussed the issue many times. In the end, we argue that the practical implementation of p-value scoring can be hindered by sample sizes (a tiny difference can be statistically significant in a large dataset) and number of categorical values of the datasets at hand. Specifically, categorical variables, such as those using ICD-9 or PheWAS codes, can have thousands of values, limiting approximations such as using Chi Square Goodness of Fit tests. Therefore, we recommend that users perform statistical tests at their discretion.

In response to the Reviewer’s comment, we also conducted an additional experiment (attached) for the NHDS case study, using a Chi-Square Goodness of Fit test. We specifically sought statistical differences in the “diagcode1-phewascode” variable (principal diagnosis at discharge), which in 2008 split the NHDS

dataset into two main subgroups. We found that the distribution in subgroup >=2008 was significantly different from the reference distribution in subgroup <2008 (p-value < 2.2e-16).

We also propose another approach: checking the extremeness of the probability distribution distances rather than the data. We refer the Reviewer to our previous work in DAMI, where a probability distribution Statistical Process Control method was provided for a bach-by-batch extremeness control.

Another option would be a bootstrap procedure that involves simulating Jensen Shannon Distances (JSDs) between two pre and post random bootstrap samples taken from the complete dataset (H0). The approach might produce a bootstrap distribution of JSD, upon which the JSD between the real pre vs post could be tested for likelihood. We argue, however, that this approach would fall out of the scope of this work.

Regarding the Reviewer's second comment, we agree that compression arises when using Multi-Dimensional Scaling (MDS) on the dissimilarity matrix between the batched statistical distributions (e.g, distributions of batches at a monthly basis). The percentage of captured information varies according to the complexity of the latent statistical manifold, as well as the number of variable category values or bins. There is also variation based on whether we use classical or non-metric MDS. Therefore, based on the Reviewer's comment, we have included this information as an additional attribute in the IGTprojection class resultant from the estimation of IGT plots on the selected number of dimensions. We note that in previous experiments with non-metric MDS, the resultant "Stress" was very close to 0, but classical MDS helped to visualize the large components of change, although with more loss of information (in R cmdscale measured as Goodness of Fit).

While the previous version of EHRtemporalVariability used internally classical MDS, we now have added the possibility of choosing between classical and non-metric MDS when estimating the IGT projections, where the resultant loss of information is returned in both cases. We thank the Reviewer for the suggestion.

R2Q4) "It seems the case studies mostly focus only on one of the 4 different types of changes which is somewhat the most trivial to identify (abrupt changes). I feel it would be beneficial to include/modify case studies on detecting trends (e.g., certain type of anti-depressant / painkiller use) and seasonality (e.g., flu) for demonstrating the full capability of the package / app. I think this is also important for educating users on how to interpret the IGT plots (i.e. spot different types of patterns)."

We agree with the Reviewer that it is important to educate users on how to interpret the IGT plots. In the manuscript, we focused predominantly on abrupt changes but also described others in the three case studies, as follows: ASD-BCH case study: Figure 2 caption: "Overall, there is a trend in the distribution changes across all the entire time frame."Mortality case study: Figure 3 highlights all four types of changes, please see arrows labeled "a" (abrupt change), "b" (trend) and "c" (seasonality), while the temporal subgroups are defined by the split provided by a. (Note: we now have also highlighted outlying months due to acute flu epidemics). NHDS: the dedicated paragraph summarizes the changes, which are fully described in our previous work.

In response to the Reviewer's suggestion, we have made two additional changes. First, we have added a new section in the package Vignette related to the interpretation of temporal changes in IGT projections (http://personales.upv.es/carsaesi/EHRtemporalVariability/EHRtemporalVariability.html#interpretation-of-temporal-changes-in-igt-projections). This new section describes the different types of changes as shown by IGT projections and then shows an example using the NHDS "Diagnosis code #1 PheWAS code" variable, where the four types of changes can be ascertained. In addition, we have emphasized throughout the manuscript the relationship between our findings and the four types of changes. We were most specific in the descriptions of case study results and also referenced the new content in the Vignette.

R2Q5) "In the conclusion I would be curious to see a brief discussion/recommendation on how one could address the dataset shifts in the data sets, specially in machine learning based on their expertise. For instance, would splitting the data into separate data sets in the model training such that the data before and after the data shift used to generate two models specific to certain time period be a plausible strategy? Or would they recommend to map (e.g., ICD9 to ICD10) / renormalize the data or perhaps add an additional feature pointing to the individual subgroups (e.g., before / after shift)."

We welcome the interest of the Reviewer in our recommendation of how to address dataset shifts. There

is not a definitive answer, given that a general recommendation depends on the target data use. i.e., for a predictive model, one may think that ignoring past data before an abrupt change is a good option, since the latest data would most closely resemble future data. In fact, incremental learning approaches rely on this concept, where “forgetting mechanisms” can be introduced to regulate the manner in which past data fades from the model memory. Since we cannot predict that nature of new unobserved data, that is a well-supported hypothesis. It is, therefore, within our current investigation to include an additional feature that points to individuals subgroups, as the reviewer suggested. The feature would be equivalent to using mixed-effect models, but including the temporal factor.

We considered the suggestion of performing separate analyses for descriptive analyses, (or at least to report both the global and the separate results).

Regarding the mapping/renormalization, the mapping processes themselves can introduce temporal variability, given the possible distinct groups of terms, such as in the cases of ICD-9 yearly updates reported in the manuscript. We have included the following text in the last paragraph of results and discussion:

'In light of the changes uncovered by EHRtemporalVariability, we argue that users of the package can more accurately repurpose their data analyses. For example, in the presence of abrupt changes, one can compare the performance of predictive modelling using only the most recent temporal subgroups versus full data inclusion. The superiority of the former is supported by the hypothesis that newly observed data will more closely resemble the latest data.

In addition, incremental learning approaches can also be adopted to deal with abrupt changes and continuous trends in machine learning, as can introducing seasonal or subgroup-related effects on models. Finally, in cases of descriptive analyses, such as those in PheWAS studies, we suggest evaluating the possible effect of temporal changes in results by making separate analyses at distinct temporal subgroups, as opposed to performing more global analysis.'

We also refer the Reviewer to Table 4 in our previous work in JAMIA (https://doi.org/10.1093/jamia/ocw010) and Table 1 in our recent work in BMJ Open (http://dx.doi.org/10.1136/bmjopen-2019-034396), where specific recommendations are provided for different types of changes.

R2Q6) "On a technical note, what are the computational complexity for running the package (e.g., expected runtime on the used system configuration)? is there a limit on how many records / variables can be uploaded? It would also be great to know the size (in terms of MB / GB) of the benchmark data sets and the time it takes to upload / analyze the data in the Shiny app (in addition to the number of features and patients)."

We refer the Reviewer to the Supplementary Material, Section 3 “Performance measures,” in which most of these issues are discussed. Regarding the limits on the Shiny app, these will theoretically depend on the web server and hardware configuration of the system and on the specific size limits set on the Shiny app. These limits can be modified by the users in the Shiny app code, now set to 100MB (shiny.maxRequestSize=100*1024^2).

R2Q7) "The examples provided in the R package seem to work without major issues. The only error I got was in the following
> class(igtProj[[1]])
Error in igtProj[[1]] : this S4 class is not subsettable"

We thank the editor for pointing out the error. That was a typo, where igtProj[[1]] should be the list igtProjs[[1]]. We fixed it accordingly.

R2Q8) "Minor:
- can *by* constructed => be"

Changed accordingly.

"- to circumvent these issues => Is this actually circumventing or identifying? If the package does not do a correction it would be rather the latter, i.e. identifying the problem rather than correcting it."

Changed to: “to identify these temporal variability issues.”

"- highly coded information => Unclear what it refers to"

We have now introduced the term in the paragraph just before:

However, these approaches tend to promulgate loss of information, especially when deployed when using highly coded data, i.e., categorical variables with a particularly high number of values.

"- concern about complex variable processing => Again unclear"

Changed to:
Analyses can proceed using both the R package and Shiny app with minimum effort. Data can flow through the pipeline from its initial raw, individual-level state to the final results.

"- over 5 years => previously"

Changed accordingly.

"- machine-learning => machine learning"

Changed accordingly.

Close
