# Supplementary material for: EHRtemporalVariability: delineating temporal data-set shifts in electronic health records
Source: Gigascience. 2020 Jul 30;9(8):giaa079. doi: 10.1093/gigascience/giaa079 (PMC7391413; doi:10.1093/gigascience/giaa079)
Supplement: giaa079_Reviewer_1_Report_Original_Submission — Jing Zhao -- 1/24/2020 Reviewed [file giaa079_reviewer_1_report_original_submission.pdf]

Reviewer Report

Title: EHRtemporalVariability: delineating temporal dataset shifts in electronic health records

Version: Original Submission **Date:** 1/24/2020

Reviewer name: Jing Zhao

Reviewer Comments to Author:

The main objective of this technical note is to introduce an R package and a Shiny app that was developed to explore and identify the temporal variability of electronic health record (EHR). To achieve this goal, the authors first gave a brief introduction to the methodologies embedded in the R tools in a way that easy to understand and elucidated the functionalities of these tools. Three case studies were listed afterward. This topic is in-time for promoting the usage of EHR in clinical research. However, it could be further improved if the following comments are soundly fixed.

1. The paper only shows the IGT plot and temporal heatmap, it would be improved if the authors provide more comprehensive analysis functions and visualizations shown in the original case studies, e.g. the kinematic figures, temporal subgroup discovery and clustering validation, the variability of JSD distributions.
2. The manuscript needs careful editing for English and style. For example, the arrows and result image A1-A3 are not vertically aligned well in Figure 1; the arrow in A2 uses a lighter grey color while the meaning seems no different from others; the different border width of B2; the local version of Shiny app accepts input of three separators as comma, semicolon, and tab, however, the authors only claim the input as comma-separated values file (CSV).
3. The abbreviation of National Hospital Discharge Survey in the title of table SM3&6 is incorrect.

Level of Interest

Please indicate how interesting you found the manuscript: Choose an item.

Quality of Written English

Please indicate the quality of language in the manuscript: Choose an item.

Declaration of Competing Interests

Please complete a declaration of competing interests, considering the following questions:

- Have you in the past five years received reimbursements, fees, funding, or salary from an organisation that may in any way gain or lose financially from the publication of this manuscript, either now or in the future?

- Do you hold any stocks or shares in an organisation that may in any way gain or lose financially from the publication of this manuscript, either now or in the future?
- Do you hold or are you currently applying for any patents relating to the content of the manuscript?
- Have you received reimbursements, fees, funding, or salary from an organization that holds or has applied for patents relating to the content of the manuscript?
- Do you have any other financial competing interests?
- Do you have any non-financial competing interests in relation to this paper?

If you can answer no to all of the above, write 'I declare that I have no competing interests' below. If your reply is yes to any, please give details below.

'I declare that I have no competing interests

I agree to the open peer review policy of the journal. I understand that my name will be included on my report to the authors and, if the manuscript is accepted for publication, my named report including any attachments I upload will be posted on the website along with the authors' responses. I agree for my report to be made available under an Open Access Creative Commons CC-BY license (http://creativecommons.org/licenses/by/4.0/). I understand that any comments which I do not wish to be included in my named report can be included as confidential comments to the editors, which will not be published.

Choose an item.

To further support our reviewers, we have joined with Publons, where you can gain additional credit to further highlight your hard work (see: https://publons.com/journal/530/gigascience). On publication of this paper, your review will be automatically added to Publons, you can then choose whether or not to claim your Publons credit. I understand this statement.

Yes Choose an item.
