# Supplementary material for: EHRtemporalVariability: delineating temporal data-set shifts in electronic health records
Source: Gigascience. 2020 Jul 30;9(8):giaa079. doi: 10.1093/gigascience/giaa079 (PMC7391413; doi:10.1093/gigascience/giaa079)
Supplement: giaa079_Reviewer_2_Report_Original_Submission — Emre Guney -- 4/26/2020 Reviewed [file giaa079_reviewer_2_report_original_submission.pdf]

Reviewer Report

Title: EHRtemporalVariability: delineating temporal dataset shifts in electronic health records

Version: Original Submission **Date:** 4/26/2020

Reviewer name: Emre Guney

Reviewer Comments to Author:

The manuscript presents an EHRtemporalVariability, an R package and accompanying Shiny app, that provides several visualization functions of EHR data towards identifying changes in the distribution of features in the data. The package accepts the data as an R data frame and allows generating Information-Geometric-Temporal (IGT) plots and Data Temporal Heatmaps (DTHs) -two types of plots previously developed by the authorsâ€” that help visually detecting different type of changes in the data described as trend, abrupt change, temporal subgroup, seasonality in the manuscript. Overall, the article is well written and the package and the app are fairly explained. I list several points that need to be addressed in my opinion.

1. The introduction needs substantial language & organization revision. The authors use "this/these" in many sentences, making hard to follow what exactly being referred in each sentence. Some examples are "These efforts …", "This is similar …", "These artifacts …". Furthermore, the introduction lacks a brief description of the existing methods mentioned by the authors (refs 8-14) as well as other relevant R packages such as EHR, rEHR, cleanEHR.
2. In the introduction the manuscript refers to existing methods based on statistical process control and time-series analysis but do not provide the references for these studies. It would be crucial to also add a short text on the contribution of this study (the proposed package) and how it relates / differs from the existing work to position the contribution of the study.
3. In most case studies, the authors suggest that there was no apparent cause for the shift. It would certainly be useful to assign a confidence/statistical score (such as p-value/confidence interval) regarding the extremeness of observing the shit in concern. Moreover, as far as I understand the projection involves data compression due to the choice of the 2-3 dimensions to be displayed, it would also be useful to know the percentage of the information the visually presented plot captures (e.g., analogous to variant explained in a PCA plot).
4. It seems the case studies mostly focus only on one of the 4 different types of changes which is somewhat the most trivial to identify (abrupt changes). I feel it would be beneficial to include/modify case studies on detecting trends (e.g., certain type of anti-depressant / painkiller use) and seasonality (e.g., flu) for demonstrating the full capability of the package / app. I think this is also important for educating users on how to interpret the IGT plots (i.e. spot different types of patterns).
5. In the conclusion I would be curious to see a brief discussion/recommendation on how one could address the dataset shifts in the data sets, specially in machine learning based on their expertise. For instance, would splitting the data into separate data sets in the model training such that the data before and after the data shift used to generate two models specific to certain time period be a plausible strategy? Or would they recommend to map (e.g., ICD9 to ICD10) / renormalize the data or perhaps add

an additional feature pointing to the individual subgroups (e.g., before / after shift).
6. On a technical note, what are the computational complexity for running the package (e.g., expected runtime on the used system configuration)? is there a limit on how many records / variables can be uploaded? It would also be great to know the size (in terms of MB / GB) of the benchmark data sets and the time it takes to upload / analyze the data in the Shiny app (in addition to the number of features and patients).
7. The examples provided in the R package seem to work without major issues. The only error I got was in the following

```
> class( igtProj[[ 1 ]] )
Error in igtProj[[1]] : this S4 class is not subsettable
```

Minor:
- can *by* constructed => be
- to circumvent these issues => Is this actually circumventing or identifying? If the package does not do a correction it would be rather the latter, i.e. identifying the problem rather than correcting it.
- highly coded information => Unclear what it refers to
- concern about complex variable processing => Again unclear
- over 5 years => previously
- machine-learning => machine learning

Level of Interest

Please indicate how interesting you found the manuscript: Choose an item.

Quality of Written English

Please indicate the quality of language in the manuscript: Choose an item.

Declaration of Competing Interests

Please complete a declaration of competing interests, considering the following questions:

- Have you in the past five years received reimbursements, fees, funding, or salary from an organisation that may in any way gain or lose financially from the publication of this manuscript, either now or in the future?
- Do you hold any stocks or shares in an organisation that may in any way gain or lose financially from the publication of this manuscript, either now or in the future?
- Do you hold or are you currently applying for any patents relating to the content of the manuscript?
- Have you received reimbursements, fees, funding, or salary from an organization that holds or has applied for patents relating to the content of the manuscript?
- Do you have any other financial competing interests?

- Do you have any non-financial competing interests in relation to this paper?

If you can answer no to all of the above, write 'I declare that I have no competing interests' below. If your reply is yes to any, please give details below.

I declare that I have no competing interests

I agree to the open peer review policy of the journal. I understand that my name will be included on my report to the authors and, if the manuscript is accepted for publication, my named report including any attachments I upload will be posted on the website along with the authors' responses. I agree for my report to be made available under an Open Access Creative Commons CC-BY license (http://creativecommons.org/licenses/by/4.0/). I understand that any comments which I do not wish to be included in my named report can be included as confidential comments to the editors, which will not be published.

Choose an item.

To further support our reviewers, we have joined with Publons, where you can gain additional credit to further highlight your hard work (see: https://publons.com/journal/530/gigascience). On publication of this paper, your review will be automatically added to Publons, you can then choose whether or not to claim your Publons credit. I understand this statement.

Yes Choose an item.
